# Lysine Fatty Acylation: Regulatory Enzymes, Research Tools, and Biological Function

**DOI:** 10.3389/fcell.2021.717503

**Published:** 2021-07-22

**Authors:** Garrison Komaniecki, Hening Lin

**Affiliations:** ^1^Graduate Field of Biochemistry, Molecular, and Cell Biology, Cornell University, Ithaca, NY, United States; ^2^Department of Chemistry and Chemical Biology, Cornell University, Ithaca, NY, United States; ^3^Howard Hughes Medical Institute, Cornell University, Ithaca, NY, United States

**Keywords:** lysine fatty acylation, protein lipidation, sirtuin, HDAC, RTX toxin, NMT, myristoylation, palmitoylation

## Abstract

Post-translational acylation of lysine side chains is a common mechanism of protein regulation. Modification by long-chain fatty acyl groups is an understudied form of lysine acylation that has gained increasing attention recently due to the characterization of enzymes that catalyze the addition and removal this modification. In this review we summarize what has been learned about lysine fatty acylation in the approximately 30 years since its initial discovery. We report on what is known about the enzymes that regulate lysine fatty acylation and their physiological functions, including tumorigenesis and bacterial pathogenesis. We also cover the effect of lysine fatty acylation on reported substrates. Generally, lysine fatty acylation increases the affinity of proteins for specific cellular membranes, but the physiological outcome depends greatly on the molecular context. Finally, we will go over the experimental tools that have been used to study lysine fatty acylation. While much has been learned about lysine fatty acylation since its initial discovery, the full scope of its biological function has yet to be realized.

## Introduction

Post-translational modification of proteins is a key biological regulatory mechanism that impacts every aspect of life. One class of post-translational modifications is protein lipidation which involves the attachment of hydrophobic moieties such as fatty acyl groups, isoprenoid lipids, or cholesterol (Jiang et al., 2018). These hydrophobic species partition into the lipophilic environment of cellular membranes, bringing the modified protein to the membranes (Resh, 1999). N-terminal glycine myristoylation and cysteine palmitoylation and prenylation are well studied forms of protein lipidation known to regulate biological processes from development and cancer to inflammation and microbial pathogenesis (Resh, 2006; Schlott et al., 2018; Wang et al., 2021). In comparison, fatty acylation of lysine residues is under-examined. However, recent progress in the understanding of the enzymes that regulate lysine fatty acylation and the effect of the modification on substrates has opened the door to exciting new research directions.

**FIGURE 1:**
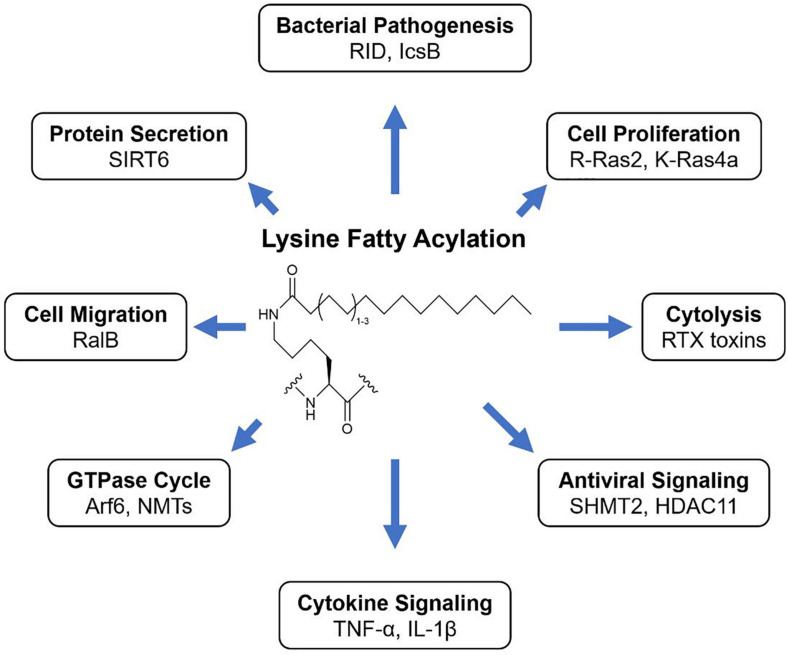
KFA is the modification of lysine side chains with long fatty acyl groups. How KFA affects a modified substrate is not always understood but known outcomes of KFA are diverse.

Lysine fatty acylation (KFA) is the addition of long-chain fatty acyl groups to lysine side chains via amide bonds. Myristoylation (C_14_) and palmitoylation (C_16_) are the most common forms of KFA, but the identity of the endogenous acyl group is often unknown making fatty acylation a more inclusive and general description (Figure 1). KFA of mammalian proteins was first discovered by Bursten et al., 1988 when studying the membrane affinity of IL-1α. Since this initial discovery, seven other human proteins and an entire class of bacterial proteins were identified to be modified by KFA. In addition, several proteins of both human and bacterial origin were found to be able to add or remove this modification. These discoveries, along with the tools that make them possible, will be covered in this review.

## Fatty Acyl Lysine Hydrolases

To date, all enzymes known to have KFA hydrolase activity fall into the lysine deacetylase (KDAC) family of proteins. This includes both Zn^2+^-dependent histone deacetylases (HDACs) and the NAD^+^-dependent sirtuins (SIRTs). Archaebacterial sirtuins Sir2Af1 and Sir2Af2 from the archaea *Archaeoglobus fulgidus*, were also reported to be able to remove KFA, but there are no known substrates for this activity (Ringel et al., 2014). The KFA hydrolases discussed below were originally assumed to only remove acetyl groups so it is possible that some of the bacterial deacetylases could also have KFA hydrolase activity (Gregoretti et al., 2004). Sir2A from the malaria parasite *Plasmodium falciparum* has also been found to have KFA hydrolase activity, but again no substrates for this activity have been identified (Zhu et al., 2012). Human enzymes HDAC8 and HDAC11 along with SIRT1, 2, 3, 6, and 7 can all remove KFA *in vitro* (Table 1). We will highlight the enzymes with known endogenous substrates for this activity in order of their discovery.

**TABLE 1:** Enzymes that regulate lysine fatty acylation.

	Name	Species	*k_*cat*_/K_*M*_* (s^–1^/M^–1^)	Known Substrates	References
Lysine fatty-acyl transferases	RtxC Family	Many gram negative bacteria	NA	RtxA toxins	Benz, 2020
	RID	*V. cholerae*	NA	RhoA-family GTPases	Zhou et al., 2017
	IcsB	*S. flexneri*	NA	Several—see citation.	Liu et al., 2018
	NMT1, NMT2	Human	NMT1: 144 ^*a*^ NMT2: 133 ^*a*^	Arf6	Dian et al., 2020; Kosciuk et al., 2020
Lysine fatty-acyl hydrolases	HDAC8	Human	120 ^*b*^	NA	Aramsangtienchai et al., 2016
	HDAC11	Human	1.54 × 10^4^ ^*b*^	SHMT2	Cao et al., 2019
	SIRT1	Human	1.44 × 10^5^ ^b^	NA	Gai et al., 2016
	SIRT2	Human	7.4 × 10^4^ ^*b*^	K-Ras4a, RalB, Arf6	Jing et al., 2017; Spiegelman et al., 2019
	SIRT3	Human	2.51 × 10^5^ ^*b*^	NA	Gai et al., 2016
	SIRT6	Human	1.4 × 10^3^ ^*b*^	TNF-α, R-Ras2	Jiang et al., 2013; Zhang et al., 2017
	SIRT7	Human	> 167 ^*c*^	NA	Tong et al., 2017

*^a^Myristoylation of Arf6 G2A peptide. Analysis using HPLC.*

*^b^Demyristoylation Myr-H3K9 peptide. Analysis using HPLC.*

*^c^Demyristoylation Myr-H3K9 peptide in the presence of rRNA. Analysis using HPLC.*

### SIRT6

The first mammalian protein identified to hydrolyze KFA is SIRT6 (Jiang et al., 2013). SIRT6 is involved in several physiological processes such as regulating immune signaling and suppressing tumorigenesis (Chang et al., 2020). SIRT6 is recruited to chromatin by DNA double strand breaks and by transcription factors such as HIF-1α to remodel chromatin and regulate gene expression (Zhong et al., 2010; Toiber et al., 2013). SIRT6 is best characterized as a lysine deacetylase. Targets of SIRT6 deacetylase activity include histone H3 and GCN5, which represses NF-kB levels and regulates glucose production, respectively (Michishita et al., 2008, 2009; Kawahara et al., 2009; Zhong et al., 2010; Dominy et al., 2012). However, *in vitro* SIRT6 deacetylase activity is relatively weak compared to other sirtuin family members, raising the possibility of additional enzymatic activities (Pan et al., 2011).

**FIGURE 2:**
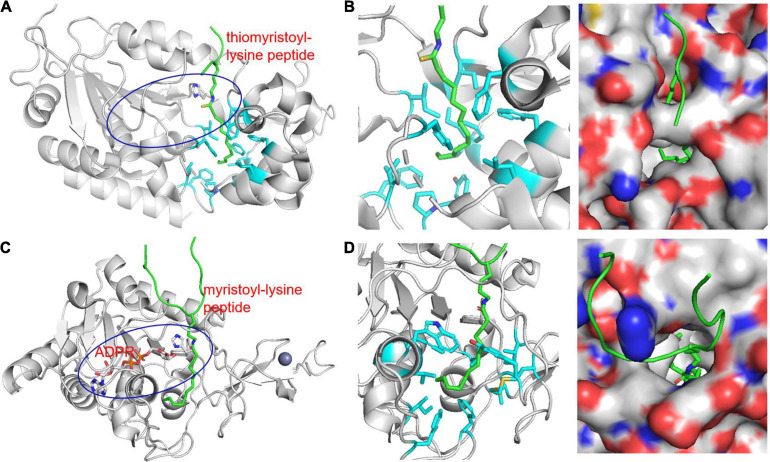
Structure of KFA hydrolases SIRT2 (**A,B**, PDB 4R8M) and SIRT6 (**C,D**, PDB 3ZG6) highlighting hydrophobic pockets that accommodate long-chain fatty acyl groups **(B,D)**. **(A)** Overall structure of SIRT2 in complex with a thiomyristoyl-lysine peptide. The peptide is shown in green. The blue oval highlights the overall active site of SIRT2 and the catalytic histidine residue is shown in stick representation. **(B)** Zoom-in view of the SIRT2 hydrophobic pocket that accommodates the myristoyl group. The hydrophobic side chains are shown in cyan stick representations. A surface representation showing the hydrophobic pocket in a slightly different orientation is also shown. **(C)** Overall structure of SIRT6 in complex with a myristoyl-lysine peptide. The peptide is shown in green. The blue oval highlights the overall active site of SIRT6 and the catalytic histidine residue is shown in stick representation. **(D)** Zoom-in view of the SIRT6 hydrophobic pocket that accommodates the myristoyl group. The hydrophobic side chains are shown in cyan stick representations. A surface representation showing the hydrophobic pocket in a different orientation is also shown.

Jiang et al. (2013) explored alternative SIRT6 deacylation activities using various acyl-lysine peptides. Like previous studies, SIRT6 had very little deacetylation activity on peptide substrates *in vitro*. However, SIRT6 was able to efficiently hydrolyze octanoyl, myristoyl, and palmitoyl lysine peptides. A SIRT6 structure was obtained by co-crystallization with a myristoyl-lysine peptide revealing a hydrophobic groove in which bound the myristoyl group (Figure 2). Interestingly, free fatty acids were found to activate SIRT6 deacetylase activity but to inhibit deKFA activity (Feldman et al., 2013). Together, these observations suggest that binding of fatty acyl groups, whether free or on a lysine, to the acyl pocket of SIRT6 may activate SIRT6.

Given the reported tumor suppressor activity of SIRT6, it is attractive to imagine compounds that could selectively activate SIRT6 through interactions with the hydrophobic groove. Indeed, multiple studies have identified compounds able to activate SIRT6 deacetylation activity (You et al., 2017, 2019; Tenhunen et al., 2021). Crystal structures consistently reveal SIRT6 activating compounds bound in the hydrophobic groove. It is therefore unsurprising that when tested in demyristoylation assays these compounds act as inhibitors (You et al., 2017, 2019). SIRT6 deacetylation inhibitors have also been developed and have been shown to have potential efficacy in type II diabetes and multiple sclerosis models (Parenti et al., 2014; Sociali et al., 2017; Ferrara et al., 2020). These inhibitors were not tested against SIRT6 deKFA activity so what role SIRT6 deKFA activity has in these contexts is unclear. Thiomyristoyl peptides can inhibit SIRT6 deKFA by taking advantage of SIRT6 activity to generate a covalent stalled intermediate (He et al., 2014). Future SIRT6 inhibitors could use this as a starting point for more potent compounds. Such an approach has proven successful for SIRT2 inhibitors as will be discussed below.

### SIRT2

SIRT2 was the next enzyme found to have endogenous substrates for its deKFA activity (Jing et al., 2017; Spiegelman et al., 2019; Kosciuk et al., 2020). SIRT2 has been extensively studied due to its diverse physiological roles and its potential as a therapeutic target for certain cancers and neurological disorders (Liu et al., 2019; Wang et al., 2019; Chen et al., 2020). In cancer, SIRT2 can play both a tumor promoting and tumor suppressing role (Chen et al., 2020). For instance, SIRT2 can promote breast cancer development by deacetylating Slug and aldehyde dehydrogenase 1A1 (ALDH1A1) (Zhao et al., 2014; Zhou et al., 2016). On the other hand, SIRT2 can reduce the activity of peroxiredoxin-1 (Prdx-1) through deacetylation which leads to breast cancer cells accumulating reactive oxygen species (ROS) and becoming less viable (Fiskus et al., 2016). Regardless, inhibition of SIRT2 leads to degradation of c-Myc and reduced growth in a broad variety of cancer cells, demonstrating its efficacy as a drug target (Jing et al., 2016).

Unlike SIRT6, SIRT2 has strong *in vitro* deacetylase activity and has a plethora of reported deacetylase targets (Feldman et al., 2013; Wang et al., 2019). While SIRT2 is a well-established deacetylase, it was also found to be able to hydrolyze lysine myristoylation with comparable efficiency to acetylation (Feldman et al., 2013; Teng et al., 2015). Similar to SIRT6, structural analysis of SIRT2 crystalized with a myristoyl-lysine peptide revealed a hydrophobic pocket that can readily accommodate a fatty acyl group (Figure 2; Teng et al., 2015).

SIRT2 inhibitors are numerous and diverse. Inhibitors of SIRT2 can be broadly categorized into two classes: activity-based and non-activity-based. Activity-based SIRT2 inhibitors usually have a peptide backbone and contain a thioacyl moiety that reacts with NAD^+^ in the SIRT2 active site to form a covalent stalled intermediate. Non-activity-based SIRT2 inhibitors function through a more typical manner by binding tightly at or near the active site. Direct comparison of SIRT2 inhibitors revealed that non-activity-based inhibitors AGK2, SirReal2, and Tenovin-6 can inhibit *in vitro* SIRT2 deacetylase activity, but not deKFA activity. Activity-based inhibitor TM was able to inhibit both activities *in vitro* (Spiegelman et al., 2018), but not much deKFA activity in cells. However, simultaneous inhibition of SIRT2 deacetylase and deKFA activity can be achieved with a proteolytic targeting chimera (PROTAC) strategy to selectively degrade SIRT2 (Schiedel et al., 2018; Hong et al., 2020). Crystal structures of inhibitors bound SIRT2 have been solved for several inhibitors (Rumpf et al., 2015; Mellini et al., 2017; Moniot et al., 2017; Hong et al., 2019). A recurring theme in these structures is the contribution of residues in the SIRT2 hydrophobic pocket for interaction with the inhibitors. While SIRT2 inhibitors have yet to be used in a clinical setting, cellular and mouse studies have yielded encouraging results for the use of SIRT2 inhibitors in treating disease. As mentioned above SIRT2i reduced growth of numerous different cancer cell lines (Jing et al., 2016). SIRT2i has also been shown to decrease α-synuclein toxicity in Parkinson’s disease models (Outeiro et al., 2007). The exact mechanism of action for this effect is still unclear and there is some debate about the causative or protective role for SIRT2 in Parkinson’s disease (Liu et al., 2019).

### HDAC11

The most recent enzyme found to hydrolyze KFA is HDAC11 (Kutil et al., 2018; Moreno-Yruela et al., 2018; Cao et al., 2019). HDAC11 is the newest member of the HDAC family and is the only class IV HDAC in humans. Since its discovery, HDAC11 has been found to play a role in neuronal function, immune regulation, and metabolic homeostasis (Bagchi et al., 2018; Sun et al., 2018a,b; Yanginlar and Logie, 2018; Nunez-Alvarez and Suelves, 2021; Yang et al., 2021). In addition, HDAC11 is overexpressed in several cancers and silencing HDAC11 can cause cell death in some cancer cell lines (Deubzer et al., 2013; Thole et al., 2017).

In *in vitro* studies, HDAC11 lacks detectable deacetylation activity. Instead, HDAC11 is highly active toward KFA modified substrates (Kutil et al., 2018; Moreno-Yruela et al., 2018; Cao et al., 2019). HDAC11 kinetics are similar to SIRT2 in hydrolyzing KFA, but HDAC11 is far more selective toward this unique modification. Unfortunately, no crystal structure has been obtained for HDAC11. HDAC11 modulation has been shown to affect acetylation of several proteins, but catalytic dead HDAC11 mutants were not utilized in any of these studies so whether or not HDAC11 has any bona-fide deacetylation substrates remains unclear (Glozak and Seto, 2009; Wang et al., 2017; Gong et al., 2019; Yuan et al., 2019). HDAC11 is reported to interact with HDAC6 so it is possible that changes in acetylation following HDAC11 modulation occur indirectly through HDAC6 or with the help of some unidentified cofactor or interacting partner that is lost in purification of HDAC11 for *in vitro* assays (Gao et al., 2002). Further work is necessary to determine whether and how HDAC11 regulates protein acetylation.

Compared to SIRT6 and SIRT2, HDAC11 is understudied. The number of published inhibitors reflects this. A common strategy for creating inhibitors for Zn^2+^-dependent HDACs is to design a molecule that can chelate Zn^2+^ and has isoform specific interactions with residues surrounding the active site. This strategy was successfully employed in the design of SIS17, with a fatty acyl moiety which likely interacts with HDAC11 in a similar manner to a KFA substrate (Son et al., 2019). Another HDAC11 inhibitor, FT895, has shown promising anti-cancer activity in lung adenocarcinoma cells by suppressing Sox2 expression (Martin et al., 2018; Bora-Singhal et al., 2020). Additionally, a natural product garcinol was shown to inhibit HDAC11 selectively (Son et al., 2020). Garcinol has several reported biological activities. Although the relevance of HDAC11 inhibition in garcinol’s various biological activities is unclear, it is interesting to note that biological effects of garcinol in mouse models share some commonality with HDAC11 knockout (Son et al., 2020). Given HDAC11’s substrate specificity for KFA hydrolysis, use of HDAC11-specific inhibitors could help illuminate the role of KFA in a biological context.

## Lysine Fatty Acyl Transferases

**FIGURE 3:**
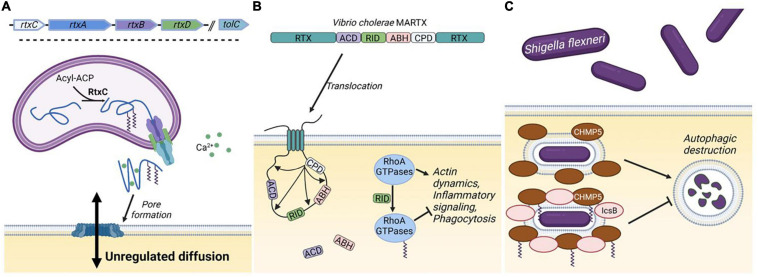
Bacterial KFA transferases. **(A)** The genetic structure for a generic RTX toxin operon includes five genes. The RtxA toxin is modified with KFA by RtxC and secreted through a transmembrane spanning pore consisting of RtxB, RtxD, and TolC. In the extracellular space, RtxA binds Ca^2+^ with characteristic nonapeptide repeats and associates with mammalian cell membranes. Oligomerization of RtxA forms large lytic pores in the membrane. **(B)** The structure of the *Vibrio cholerae* MARTX toxin is illustrated. Interior domains vary from species to species, but the terminal RTX domains in a MARTX toxin allows for translocation through the cell membrane. Once in the cell the cysteine protease domain (CPD) is activated to cleave the toxin at several points releasing the other domains: the actin crosslinking domain (ACD), the α/β hydrolase domain (ABH), and the Rho-inactivating domain (RID). RID modifies RhoA-family GTPases with KFA to suppress multiple cellular processes. **(C)** During part of the pathogenesis of *Shigella flexneri*, bacteria reside in an intracellular vacuole that can be destroyed through autophagy involving CHMP5. IcsB blocks autophagic destruction by catalyzing KFA on CHMP5. Figure created with BioRender.com.

One of the major impediments to studying KFA is the identification of human KFA transferase enzymes. Knowledge of how proteins acquire KFA would be invaluable in understanding the purpose of this modification. For instance, some bacterial toxins with known physiological importance take advantage of this activity during pathogenesis to promote infection (Figure 3). Although human lysine fatty acyl transferases are known, the acyl transferases for many of the reported KFA-modified proteins are still unknown. It is possible that such enzymes do exist but require more future effort to identify. However, an alternative explanation for the presence of endogenous KFA is that this modification simply arises from an S-to-N transfer. In this model a free lysine acts as a nucleophile to steal a fatty acyl group from a palmitoylated cysteine or palmitoyl-CoA. An amide bond is more stable than a thioester bond, making this reaction thermodynamically favorable. Several KFA transferases have been characterized and are reviewed below in order of discovery.

### RtxC Proteins

Several species of bacterial pathogens employ a class of secreted toxin proteins known as Repeats in ToXin (RTX) toxins. RTX toxins function in various ways, but typically act as cytolysins by forming pores in the plasma membrane of mammalian cells (Benz, 2016). Structures in the RTX toxin operon vary, but typically consist of four genes: *rtxA-D*. *rtxA* encodes the actual toxin which is secreted through a type one secretion system encoded by *rtxB, rtxD*, and a third gene, *tolC*, located elsewhere on the bacterial chromosome. RtxA is synthesized as a protoxin that is activated by fatty acylation on one or two highly conserved lysine residues catalyzed by RtxC (Figure 3A; Benz, 2020).

The best characterized RtxC member is HlyC, which catalyzes KFA on the *Escherichia coli* hemolysin toxin HlyA. Initial characterization of the HlyA toxin revealed that HlyC activates proHlyA in a manner dependent on the acylated acyl carrier protein (acyl-ACP) (Issartel et al., 1991). Follow-up studies determined through mass spectrometry that HlyC activates HlyA by directing fatty acylation of two internal lysines (Stanley et al., 1994). While the majority of HlyA lysine acylation is myristoylation (C14), HlyC apparently has some flexibility in substrate preference as saturated C15 and C17 acylation was also detected at appreciable amounts (Lim et al., 2000). Similar findings were made for the *Bordetella pertussis* CyaA toxin which is palmitoylated on a single lysine (Hackett et al., 1994). Interestingly, when recombinantly expressed with its cognate acyl transferase CyaC in *E. coli*, CyaA is palmitoylated on a second lysine (Hackett et al., 1995). This reveals that the number of lysines and the nature of the transferred acyl chain vary by each unique enzyme as well as the species background.

More detailed information of RtxC enzymology was revealed using HlyC (Trent et al., 1999; Worsham et al., 2001). HlyC acylation of proHlyA occurs through a ping-pong mechanism involving two steps. In the proposed model, the acyl group from acyl-ACP is first transferred to His23 to form a covalent acyl-HlyC intermediate. This His is conserved throughout RtxC proteins. Then, the acyl group is transferred to the lysine residues on proHlyA. Ser20 is also important for optimal activity. While detailed enzymology has not been carried out for many RtxC proteins, the enzymatic mechanism is likely to be similar due to the conservation of relevant catalytic residues. Indeed, the analogous active site His and Ser in CyaC are necessary for its activity (Basar et al., 2001).

Structural information for RtxC proteins is sparse but revealing. Despite unidentifiable sequence homology, ApxIC, the RtxC from *Actinobacillus pleuropneumoniae*, has conspicuous structural homology to the Gcn5-like N-acetyl transferase (GNAT) superfamily (Greene et al., 2015). GNAT proteins are well established acyl-transferases that use acyl-CoA as an acyl donor (Salah Ud-Din et al., 2016). The ApxIC structure is differentiated from other GNAT proteins by the lack of elements that typically interact with CoA, explaining why characterized RtxC proteins do not utilize acyl-CoA as the acyl donor. The conserved active site residues reside within a deep surface groove. While further structural information is needed, this study, along with the fact that RtxC proteins are well conserved, opens the door for further understanding of these unique enzymes and raises the possibility of designing inhibitors to block toxin function.

### RID

There is a subset of multifunctional RtxA toxins that are much larger called Multifunctional Autoprocessing Repeats in ToXin (MARTX) toxins. MARTX toxins contain the trademark non-apeptide repeats in both the N- and C-terminus, which forms a pore in the cell membrane that translocates the interior portion of the toxin encoding a modular array of effector domains into the cytoplasm (Satchell, 2007). While the number and type of interior domains varies, a cysteine protease domain (CPD) is universally conserved in MARTX toxins. Once internalized, the CPD is activated and proteolytically cleaves the toxin at several points, releasing additional effector domains into the cell (Satchell, 2011). One of these effectors present in multiple species was dubbed the Rho inactivation domain (RID).

RID is present in several known MARTX toxins (Satchell, 2007). The best characterized RID domain is the one in the *Vibrio cholerae* toxin, MARTX_*Vc*_ (Figure 3B). MARTX_*Vc*_ is known to induce cell rounding through an actin crosslinking domain (ACD) that covalently links actin monomers, preventing actin polymerization (Cordero et al., 2006). However, cell rounding was still observed when this domain was knocked out. Follow up studies determined that this occurred through RID, which substantially decreases the amount of GTP-bound Rho GTPases Rho, Rac, and Cdc42 (Sheahan and Satchell, 2007). The mechanism by which RID functions was not known until a structure of the *Vibrio vulnificus* RID domain was solved (Zhou et al., 2017). Clues from this structure allowed the authors to determine that RID catalyzes palmitoylation on lysines in the polybasic region of RhoA-family GTPases. This activity was dependent on C-terminal prenylation of the GTPases and could utilize palmitoyl-CoA as a acyl donor. Small GTPases are activated by guanine nucleotide exchange factors (GEFs) by stimulating the release of GDP, to allow the binding of GTP. RID-catalyzed KFA on Rac1 inhibits its interaction with GEFs, but how KFA prevents GEF interaction is unclear.

Both the ACD and RID from MARTX_*Vc*_ lead to an obvious cell rounding phenotype by preventing actin dynamics. It is reasonable to wonder why *V. cholerae* would evolve to have two domains to carry out the same role. Woida and Satchell (2020) recently discovered that RID and a third domain in the MARTX_*Vc*_, a α/β hydrolase (ABH) domain, function to suppress proinflammatory signaling. Cytoskeletal collapse caused by ACD activates mitogen-activated protein kinase (MAPK) signaling, leading to upregulation and enhanced secretion of proinflammatory cytokines. RID inactivation of Rac1 blocks MAPK signaling and subsequent cytokine secretion (Figure 3B). RhoA-family GTPases also have other functions in immune processes, so RID could be playing a broad immunosuppressive function to prevent *V. cholerae* clearance by leukocytes (Biro et al., 2014; Bros et al., 2019; Guo, 2021).

### IcsB

The *Shigella flexneri* toxin IcsB is another enzyme identified to catalyze KFA (Liu et al., 2018). *Shigella* are gram negative bacteria that can colonize the intestinal epithelium through a complicated mechanism with the aid of toxin effectors secreted by a type three secretion system (Parsot, 2009). Following ingestion, *Shigella* induce endocytosis by M cells in the epithelium of the colon (Wassef et al., 1989). Endocytosed *Shigella* are transferred to resident macrophages where they employ a barrage of secreted effectors to escape the vacuole and replicate in the cytosol of the macrophage (Zychlinsky et al., 1992). This leads to lysis of the macrophage and dissemination of bacterial progeny. One of the critical effectors that enables this mode of infection is IcsB.

IcsB promotes infection in several ways including preventing autophagic destruction and promoting cell lysis (Allaoui et al., 1992; Ogawa et al., 2005). How IcsB functions to modulate multiple cellular processes had been unclear, but it was predicted to be an enzyme through bioinformatic analysis (Pei and Grishin, 2009). IcsB was found to be a potential homolog of RID and ectopic expression of IcsB was found to disrupt the actin cytoskeleton. In line with these observations, IcsB was also found to catalyze KFA on lysines in the polybasic region of Rho GTPases (Liu et al., 2018). The eighteen-carbon stearoyl-CoA seems to be the preferred acyl donor and prenylation of Rho GTPases is also necessary for IcsB activity. Proteomic analysis identified numerous IcsB substrates in addition to Rho GTPases. KFA on one of these substrates, CHMP5, was identified to inhibit the autophagic destruction of *S. flexneri* thus providing a mechanism for sustained intercellular survival (Figure 3C).

While RID and IcsB are both bacterial toxins that catalyze KFA of host substrates, they differ in a few key aspects. IcsB transfected cells exhibit cell rounding whereas *S. flexneri* infected cells do not. Cells introduced to RID or infected with *V. cholerae* both exhibit cell rounding. This discrepancy is in part due to the presence of the *V. cholerae* ACD as well as to the observation that *S. felxneri* activate actin polymerization for part of their pathogenesis. Additionally, while no proteomic search for substrates has been done, RID is only known to modify Rho GTPases while IcsB can modify many other substrates in addition to Rho GTPases. What role KFA plays on these other substrates is an area for future study.

### NMT1 and NMT2

N-myristoyltransferases (NMTs) have long been known to transfer a myristoyl group from myrsitoyl-CoA to the α-amine of an N-terminal glycine following cleavage of the initiator methionine (Towler et al., 1987). This modification happens co-translationally or after proteolytic events that result in a free N-terminal glycine such as caspase cleavage (Farazi et al., 2001; Yuan et al., 2020). There are no known enzymes that can hydrolyze N-terminal myristoylation and as such it is thought to be irreversible. Like KFA, N-terminal myristoylation increases a protein’s affinity for membranes. Knocking out NMT is embryonically lethal in mice and fruit flies (Ntwasa et al., 2001; Yang et al., 2005). NMT levels are elevated in several cancers and NMT inhibition has advanced to clinical trials for the treatment of NMT-deficient blood cancers (Selvakumar et al., 2007; Berthiaume and Beauchamp, 2018). Additionally, several viruses utilize host NMTs for their replication and infectivity raising the possibility of using NMT inhibitors as an antiviral agent (Mousnier et al., 2018).

**FIGURE 4:**
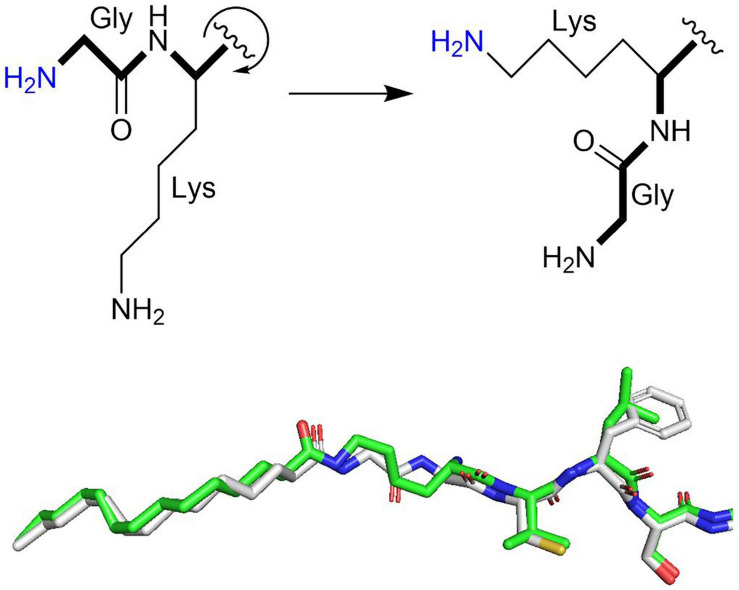
Model for NMT KFA transferase activity. Rotation around the peptide backbone (bold) positions the amine of a lysine side chain similarly to the amine of an N-terminal glycine. Both are primary amines that can rotate freely during NMT catalysis. The superimposed myristoyl-lysine (green stick representation) and myristoyl-glycine (white stick representation) in human NMT2 (PDB 6PAU) and NMT1 (PDB 5O9V) structures overlap nicely, supporting this model.

Because NMTs catalyze the fatty acylation of a primary amine, they can do the same chemistry as a potential KFA transferase. Indeed, two groups found that the human NMT1 and NMT2 can catalyze KFA on lysines toward the N-terminal of a peptide (Dian et al., 2020; Kosciuk et al., 2020). The proposed mechanism for this reaction requires rotation around the peptide bond to present the lysine ε-amine into the active site typically occupied by the α-amine of glycine (Figure 4). Both amines can freely rotate during NMT catalysis and myristoyl-glycine and myristoyl-lysine are similarly positioned in the active site of NMT structures, supporting the proposed activity (Figure 4). The precise sequence requirements for this activity are still being clarified, but it is clear that this activity is strongest when lysine is closer to the N-terminus. While glycine is the preferred substrate, KFA is efficiently catalyzed on a lysine that is right after the glycine, especially when the N-terminal amine is blocked. KFA transferase activity is decreased when an extra glycine is added before the modified lysine and is absent when two additional glycines are inserted. This narrows the scope of potential substrates for NMT KFA transferase activity. Peptide experiments indicate that a good KFA transferase substrate for NMTs has a small amino acid at position two, a lysine at position three, a serine at position six, and lysine at position seven. This was confirmed when Arf6 was identified to be a substrate of NMT KFA transferase activity (Kosciuk et al., 2020). The Arf6 N-terminal sequence following initiator methionine cleavage is GKVLSKIF. Arf6 can be doubly myristoylated at both G2 and K3. It was proposed that NMTs can accommodate both myristoyl groups when one is inserted into a solvent channel in the protein structure. While Arf6 is currently the only known substrate for NMT KFA transferase activity, it is feasible that additional proteins with N-termini similar to Arf6 could also be modified (Kosciuk and Lin, 2020).

## Functions of Lysine Fatty Acylation

**FIGURE 5:**
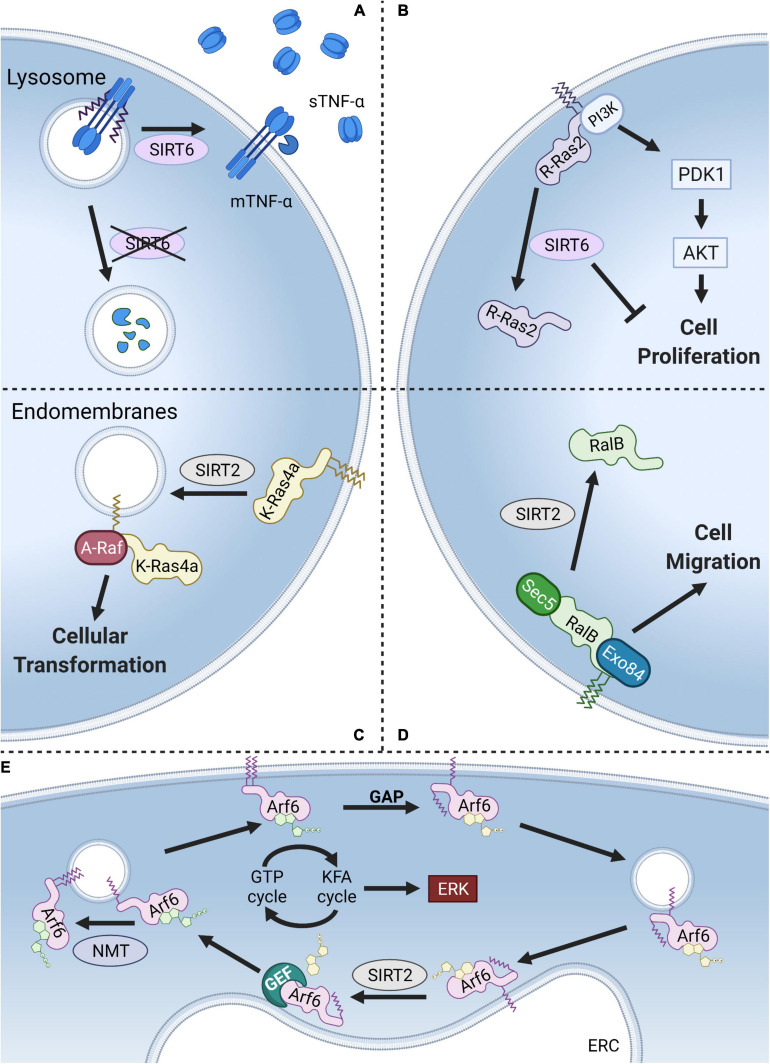
Models of KFA regulation. **(A)** KFA on TNF-α directs it to the lysosome for degradation. SIRT6 hydrolyzes TNF-α KFA leading to higher levels on the plasma membrane and increased secretion of cleaved, soluble TNF-α. **(B)** KFA on the polybasic region of R-Ras2 results in increased plasma membrane localization, PI3K interaction, AKT signaling, and cell proliferation. SIRT6 hydrolyzes R-Ras2 KFA, releasing it from the membrane and decreasing cell proliferation. **(C)** K-Ras4a with KFA preferentially localizes at the plasma membrane. SIRT2 hydrolyzes K-Ras4a KFA and promotes its localization to endomembranes where K-Ras4a interacts with the signaling kinase A-Raf leading to downstream cellular transformation. **(D)** KFA on RalB also directs it to the plasma membrane to interact with Sec5 and Exo84 and promote cell migration. SIRT2 hydrolyzes RalB KFA, decreasing plasma membrane localization. **(E)** NMT-catalyzed KFA on K3 of Arf6 increases plasma membrane localization. GTP hydrolysis at the plasma membrane aided by a GTPase activating protein (GAP) causes Arf6 N-terminal glycine myristoylation to be sequestered. Arf6 is then trafficked to the endocytic recycling compartment (ERC) where SIRT2 hydrolyzes KFA and Arf6 is reloaded with GTP. NMT then starts the cycle again. Thus, a KFA and deKFA cycle promotes Arf6 activation, which in turn promotes ERK signaling. Figure created with BioRender.com.

A diverse set of proteins has been identified to have KFA (Table 2). How exactly KFA affects the modified protein is often unclear, but a common theme is a change in membrane affinity (Figure 5). If a protein is otherwise soluble, KFA can lead to membrane localization as is the case for IL-1α or SHMT2. For proteins that already have membrane targeting elements, KFA can change which cellular membranes they are localized to. This is the case for several small GTPases which are targeted to membranes through electrostatic interactions and other lipid modifications. In this section we review proteins known to have KFA, how KFA on the protein was found, and what is known about how KFA affects protein function. Not discussed in this section are the substrates for RID and IcsB toxins which are examined above or in other publications (Zhou et al., 2017; Liu et al., 2018).

**TABLE 2:** Proteins with lysine fatty acylation.

Name	Species	KFA transferase	KFA hydrolase	Effect of KFA	References
IL-1α	Human	NA	NA	NA	Bursten et al., 1988; Stevenson et al., 1993
Aquaporin-0	Human, Bovine	NA	NA	NA	Schey et al., 2010
TNF-α	Human	NA	SIRT6	Decreased TNF-α secretion	Stevenson et al., 1992; Jiang et al., 2016
R-Ras2	Human	NA	SIRT6	Increased R-Ras2 activity and cell proliferation	Zhang et al., 2017
K-Ras4	Human	NA	SIRT2	Decreased interaction with A-Raf and cell proliferation	Jing et al., 2017
H-Ras	Human	NA	NA	NA	Jing et al., 2017
RalB	Human	NA	SIRT2	Increased RalB activity and cell migration	Spiegelman et al., 2019
Arf6	Human	NMT1/NMT2	SIRT2	Promotes Arf6 GTPase cycle	Kosciuk et al., 2020
SHMT2	Human	NA	HDAC11	Reduced ubiquitination and enhanced signaling of INFαR1	Cao et al., 2019
RhoA GTPases	Human	RID	NA	GTPase inactivation; defect in actin polymerization; reduced inflammatory signaling	Zhou et al., 2017; Woida and Satchell, 2020
CHMP5*	Human	IcsB	NA	Defect in *S. flexneri* autophagic destruction	Liu et al., 2018
RTX toxins	Many gram negative bacteria	RtxC	NA	Increased binding to β_2_-integrins; promotion of oligomerization	Benz, 2020

*CHMP5 is one of many identified IcsB substrates. For a complete list see Liu et al., 2018.

### IL-1α

Interleukins (ILs) are a family of secreted proteins used in cell-to-cell communication that regulate inflammatory signaling. The IL-1 family of proteins contains 11 different members that are synthesized as precursor proteins, requiring proteolytic processing for optimal biologic activity (Afonina et al., 2015). IL-1α and IL-1β are closely related pro-inflammatory members of the IL-1 family. Despite obvious structural similarity between the two proteins, they differ in some key respects (Priestle et al., 1989; Ren et al., 2017). While IL-1β functions exclusively as a secreted protein, IL-1α has activity both secreted and bound to the cell membrane (Kim et al., 2013). In examining its affinity for membranes, it was determined that IL-1α was doubly myristoylated on the N-terminal portion of the protein (Bursten et al., 1988; Stevenson et al., 1993). This was determined through the incorporation of radiolabeled myristic acid. Incubation of synthetic peptides with monocyte lysates identified lysines 82 and 83 as sites of myristoylation (Stevenson et al., 1993). Myristoylation was also observed for IL-1β, but to a much less extent. While the precise role KFA plays on IL-1α function is unclear, only the uncleaved IL-1α, with modified lysines present, associates with the cell membrane (Beuscher and Colten, 1988). It is not known what enzymes could regulate IL-1α KFA, but acylation in the presence of lysates raises the possibility of an unidentified KFA transferase. Membrane associated IL-1α contributes to arthritis in a mouse model, so modulating IL-1α KFA could have therapeutic applications (Niki et al., 2004).

### Aquaporin-0

Aquaporins are a family of transmembrane channel proteins that facilitate the transport of water across the plasma membrane. Aquaporin-0 (AQP0) is highly abundant in the ocular lens where it plays an important role, not only in circulating water, but as a cell to cell adhesion protein for maintenance of proper tissue structure (Mathias et al., 1997; Sindhu Kumari et al., 2015). There is very little protein turnover in lens proteins due to the loss of fiber cell organelles during differentiation, making proteins in this tissue an interesting model for aging. AQP0 is known to accumulate post-translational modifications with age (Ball et al., 2004). To determine what fatty acylations may be accumulating on AQP0, Schey et al. (2010) carried out mass spectrometric analysis of hydrophobic peptides from bovine and human lens tissue. They identified AQP0 as containing two fatty acylations: one on the N-terminal methionine and one on a highly conserved lysine. The identity of AQP0 KFA modification varies from C16 to C20 with up to 4 desaturations at ratios closely resembling the abundance of phosphoethanolamine lipids in lens membranes (Ismail et al., 2016). This suggests that either the modification is accumulating non-enzymatically or that a potential KFA transferase has limited preference in acyl group. The effect KFA has on AQP0 function is unknown, but the modified protein partitions to detergent-resistant membrane fractions. This suggests a role in membrane domain targeting.

### TNF-α

Tumor necrosis factor α (TNF-α) is a proinflammatory cytokine secreted by several immune cells. TNF-α is translated with a single transmembrane domain that is cleaved at the plasma membrane to release a soluble form that binds TNF receptors (TNFRs). TNFR1 is universally expressed on almost every cell type meaning that TNF-α signaling is both ubiquitous and tightly regulated (Locksley et al., 2001). In a follow up study to the IL-1α findings highlighted above, TNF-α was also identified to have KFA (Stevenson et al., 1992). Modification is present on two lysines, K19 and K20, at the intracellular end of the transmembrane helix. SIRT6 was found to hydrolyze KFA on TNF-α which in turn promotes its secretion (Jiang et al., 2013). KFA causes TNF-α to accumulate in the lysosome where it is degraded (Jiang et al., 2016). In this context, KFA thus serves an anti-inflammatory role by decreasing the amount of secreted TNF-α (Figure 5A). In addition to TNF-α, a proteomic study revealed that SIRT6 deKFA activity regulates the secretion of numerous other proteins (Zhang et al., 2016). Interestingly, SIRT6 deKFA activity decreases the secretion of ribosomal proteins via exosomes, but the detailed mechanism for how this happens is unknown.

### R-Ras2

The only other known endogenous substrate for SIRT6 deKFA activity is R-Ras2, a member of the Ras family of GTPases (Zhang et al., 2017). Ras GTPases are known to be targeted to cellular membranes by their C-terminal hypervariable regions through cysteine lipidation and polybasic regions. R-Ras2 was identified to have KFA in its polybasic region using mass spectrometry. Mutating a patch of four lysine residues to arginine in the polybasic region of R-Ras2 abolished KFA. Immunofluorescence analysis demonstrated that KFA enhances R-Ras2 plasma membrane localization. There, R-Ras2 exist more in the GTP-bound state, interacts with PI3K, activates Akt signaling, and upregulates cell proliferation. SIRT6 KO MEFs are more proliferative than WT, an effect which is rescued by suppressing R-Ras2 level. Together this provides a model where SIRT6 acts as a tumor suppressor by inhibiting R-Ras2 and downregulating proliferative PI3K/Akt signaling (Figure 5B).

### K-Ras4a

The first identified SIRT2 deKFA substrate was K-Ras4a (Jing et al., 2017). After the identification of R-Ras2 as a KFA substrate, Jing et al. (2017) examined other small GTPases with polybasic regions similar to R-Ras2 for potential KFA modification. These included the well-known oncoproteins H-Ras, N-Ras, K-Ras4a, and K-Ras4b. Using mass spectrometry, they identified both H-Ras, and K-Ras4a as having KFA in their polybasic region. Sirtuins with known deKFA activity were screened against both H-Ras and K-Ras4a and SIRT2 was found to remove K-Ras4a KFA. Removal of H-Ras KFA was not observed for any of the screened enzymes. The oncogene *KRAS* (which is alternatively spliced into K-Ras4a and K-Ras4b) is the most frequently mutated gene in cancer, so identifying how modifications regulate its activity is of great interest (Simanshu et al., 2017). KFA on K-Ras4a was found to inhibit intracellular puncta localization, interaction with the signaling kinase A-Raf, and downstream cellular proliferation (Figure 5C). By removing K-Ras4a KFA, SIRT2 serves a tumor promoting role.

### RalB

The identification of R-Ras2 and K-Ras4a as proteins with KFA motivated Spiegelman et al. (2019) to further expand the search further into the of Ras subfamily small GTPases. In so doing they identified RalB having KFA. Like R-Ras2 and K-Ras4a, RalB is modified with KFA in its C-terminal polybasic region. Mutating all eight lysines in this region to arginines abolished KFA signal. Screening enzymes with deKFA activity identified SIRT2 as being able to remove KFA from RalB. RalB has been identified to promote multiple cancer phenotypes (Martin et al., 2011; Guin et al., 2013; Tecleab et al., 2014). Studying the effect of RalB KFA on these phenotypes revealed that RalB KFA enhanced migration of A549 lung cancer cells but did not affect cell proliferation. KFA of RalB was also found to increase GTP loading, plasma membrane localization, and co-localization with its effector proteins Sec5 and Exo84 (Figure 5D). Contrary to the K-Ras4a mechanism, SIRT2 by removing RalB KFA, decreases RalB activation and cell migration. This juxtaposition underscores the diverse, context-dependent role of KFA and the enzymes that modulate it.

### Arf6

Arf6 is a small GTPase in the ADP-ribosylation factor (Arf) subfamily that has been found to be modified with KFA (Kosciuk et al., 2020). While members of the Ras subfamily of GTPases discussed above are anchored to membranes through lipidation and electrostatic interactions in their C-terminus, the Arf family is targeted to membranes via N-terminal glycine myristoylation catalyzed by NMTs (Gillingham and Munro, 2007). Unlike KFA, there are no enzymes that are known to hydrolyze glycine myristoylation. For Arf proteins, regulating membrane association occurs through a nucleotide-dependent conformational shift that flips the myristoyl group back into the protein to sequester it in a hydrophobic pocket (Goldberg, 1998). Unlike Arf1–5, Arf6 has a lysine in the third position of its sequence. Interestingly, K3 was found to be modified with KFA by NMT enzymes and the modification is hydrolyzed by SIRT2. NMT activity is greatest toward the active, GTP-bound, form of Arf6 where SIRT2 prefers the inactive, GDP-bound, form. This enzyme preference links a dynamic KFA cycle to the GTPase cycle. Indeed, inhibiting either SIRT2 or NMTs decreased phosphorylation of ERK, a downstream target of Arf6 activation. A cycle of dynamic KFA modification thus drives the Arf6 GTPase cycle (Figure 5E).

### SHMT2

Serine hydroxymethyltransferase 2 (SHMT2) is a key member of one-carbon metabolism. SHMT2 catalyzes the reversible conversion of serine and THF to glycine and methylene-THF in the mitochondria. High levels of SHMT2 are associated with poor patient prognosis in several cancers and inhibitors that target both SHMT2 and SHMT1 (which catalyzes the same reaction in the cytoplasm) have shown promising results in initial cellular and mouse studies (Cuthbertson et al., 2021). Additionally, SHMT2 was found to regulate immune signaling as a component of the BRISC deubiquitylase complex. The BRISC complex is known to hydrolyze K63-linked polyubiquitin chains on type I IFN receptor chain 1 (IFNαR1), a key receptor in the antiviral response. BRISC prevents degradation of the receptor and promotes recycling to the plasma membrane (Zheng et al., 2013; Rabl et al., 2019; Walden et al., 2019; Rabl, 2020). SHMT2 was identified to have KFA during a search for substrates of HDAC11 (Cao et al., 2019). KFA on SHMT2 did not affect its *in vitro* activity in converting serine and THF to glycine and methylene-THF. Instead, KFA was found to be relevant in SHMT2’s role in the BRISC complex. Increasing SHMT2 KFA, through HDAC11 knock out or knock down, increased SHMT2 localization to the endosomes/lysosomes where the BRISC complex can hydrolyze IFNαR1 ubiquitination. Correspondingly, HDAC11 KO cells had more IFNαR1 on the cell surface following IFNα treatment. Further, HDAC11 KO cells had stronger downstream signaling following IFNα stimulation and HDAC11 KO mice also had a stronger antiviral response when challenged with vesicular stomatitis virus. In this context, KFA again controls intracellular localization of the substrate protein, here leading to enhanced cellular activity of a deubiquitylase complex. Inhibiting HDAC11 may therefor enhance antiviral responses.

### RTX Toxins

The RtxA family of proteins are toxins that are secreted by many pathogenic gram-negative bacteria (Linhartova et al., 2010). As discussed above, RtxA proteins, hereby referred to as RTX toxins, are activated via lysine fatty acylation catalyzed by RtxC proteins. Once secreted, RTX toxins function in a variety of different ways. The best-known mode of action for RTX toxins is cytolysis and hemolysis through formation of pores in the cell membrane (Ostolaza et al., 2019). RTX toxins primarily target leukocytes and form pores in the cell membrane in a calcium dependent manner (Knapp et al., 2003). Hydrophobic regions toward the N-terminus are believed to interact with the target cell membrane to form cation-selective pores, leading to cell lysis (Iwaki et al., 1995; Welch, 2001). KFA is necessary for the cytotoxicity of all known pore forming RTX toxins, but the mechanism by which KFA affects RTX toxin function is still unclear. Unacylated RTX toxins from *E. coli* and *B. pertussis*, HlyA and CyaA, respectively, are both able to form pores in liposomes and planar lipid bilayers (Ludwig et al., 1996; Masin et al., 2005). At concentrations insufficient to cause cell lysis, RTX toxins bind to β_2_-integrins and activate downstream signaling pathways that lead to apoptosis (Atapattu and Czuprynski, 2007; Frey, 2019). KFA was found to increase the affinity to β_2_-integrin receptors for CyaC and the *A. actinomycetemcomitans* toxin LtxA (El-Azami-El-Idrissi et al., 2003; Balashova et al., 2009). It was reported that KFA on HlyA is important for the formation of oligomers in erythrocyte membranes, but the physiological importance of RTX toxin oligomerization is unclear (Herlax et al., 2009). It is conceivable that KFA on RTX toxins may promote localization to appropriate membrane subdomains such as lipid rafts, somehow increasing toxin function. For instance, the *K. kingae* RtxA binds cholesterol, a membrane component enriched in lipid rafts, for optimal activity and palmitoylation on cysteines is thought to target transmembrane proteins to lipid rafts (Levental et al., 2010; Osickova et al., 2018). KFA on RTX toxins could also potentially play a role in maintaining an appropriate structural confirmation. Additional studies are necessary to identify the precise mechanism by which KFA regulates RTX toxins.

## Tools for Studying Lysine Fatty Acylation

### Methods to Detect Endogenous KFA

**FIGURE 6:**
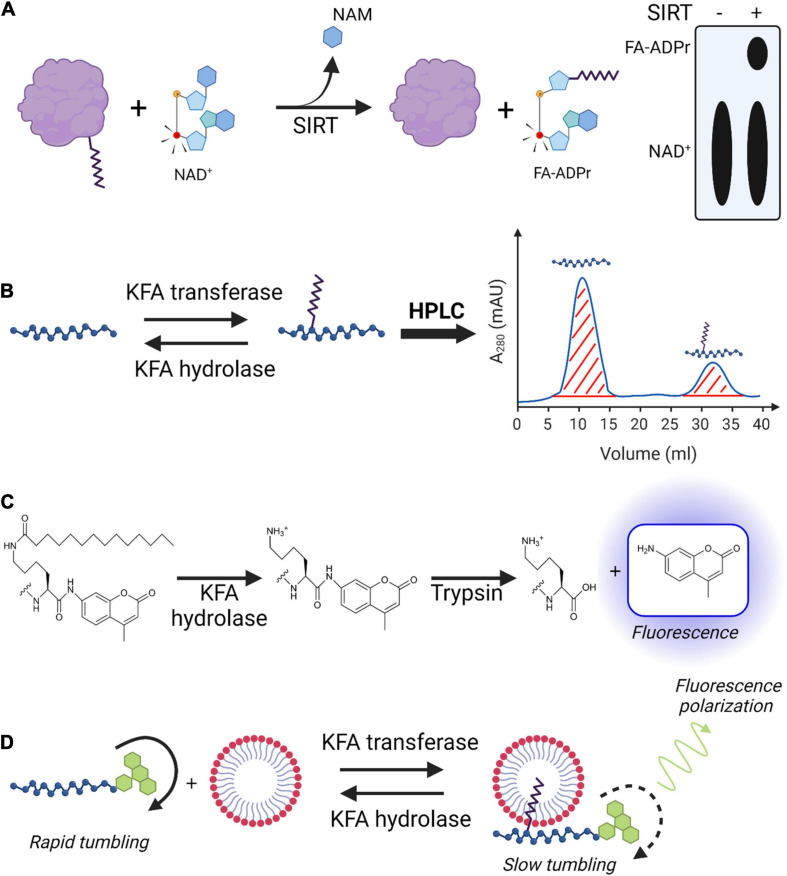
Techniques for studying KFA enzyme activity. **(A)** [^32^P]NAD^+^ sirtuin assay. ^32^P (red atom) is incorporated into NAD^+^ to be used by a sirtuin with KFA hydrolase activity. During the hydrolysis mechanism, the sirtuin transfers the fatty acyl group from a modified protein to the cofactor releasing nicotinamide (NAM) and resulting in the formation of [^32^P]fatty acyl-ADPR. TLC plates are used to separate, visualize, and quantify radiolabeled species. **(B)** HPLC analysis of KFA enzyme activity on peptides. Unmodified and KFA-modified peptides are separated with HPLC and the area of the corresponding peaks are determined for quantification. **(C)** Fluorescence-based peptide KFA hydrolase assay. KFA is hydrolyzed from a lysine immediately preceding and AMC group. Trypsin hydrolyzes the amide bond following only the unmodified lysine releasing the AMC, which then exhibits fluorescence. **(D)** Acyl-cLIP assay for KFA enzymes. KFA on a peptide modified with fluorescein increases its affinity for micellar membranes. Fluorescein has slower tumbling when bound to the bulky micelles, resulting in increased fluorescence polarization. Figure created with BioRender.com.

Research involving KFA is well suited to a chemical biology approach. The most commonly used approach when assaying KFA is the use of fatty acyl alkyne probes as discussed below. However, this approach has inherent limitations. First, one must add alkyne probes exogenously which is difficult for applications in mice or other tissue samples. Second, when used in cell cultures, a working concentration is ∼50 μM which may be sufficient to unintentionally stimulate signaling pathways involving fatty acids or to increase protein lipidation due to artificially high amounts of fatty acids. One way to assay levels of endogenous KFA is to purify a protein of interest and to attempt to identify KFA with mass spectrometry (Kosciuk et al., 2020). Mass spectrometry can directly identify the modified residue but is technically challenging due to the relatively low abundance of KFA and the hydrophobicity of the modification which makes processing samples for MS difficult. Endogenous KFA can be indirectly detected using a [^32^P]NAD^+^ TLC assay if the modification is able to be hydrolyzed by a sirtuin (Jing et al., 2017; Zhang et al., 2017; Kosciuk et al., 2020). In this assay, a protein of interest is incubated with a sirtuin that has KFA hydrolase activity and radiolabeled [^32^P]NAD^+^. Sirtuin-catalyzed lysine deacylation results in the formation of [^32^P]O-acylADPR. Fatty acyl-ADPr is drastically more hydrophobic than NAD^+^ and can be easily separated on a TLC plate. Phosphorescence detection can then be used to analyze the presence of fatty acyl-ADPR and, by extension, KFA on the protein of interest (Figure 6A). Additional tools to study endogenous KFA could be inspired by research of similar post translational modifications. Studies concerning lysine acetylation commonly employ antibodies specific to acetyl-lysine. Antibodies that could specifically bind KFA would allow for purification and analysis of endogenously modified proteins without any exogenous treatment. Additional tools for studying KFA are necessary to fully realize the full scope of biological importance.

### Enzyme Activity Assays

Assays measuring *in vitro* enzyme activity are necessary for establishing enzyme kinetics, substrate preferences, and for testing inhibitors. Once the proper cofactors and buffer conditions are determined for an enzyme of interest, measuring its activity in regulating KFA can be straightforward. Assays for RtxC enzymes indirectly measure activity by taking advantage of the hemolytic action of their cognate RtxA proteins (Bellalou et al., 1990; Linhartova et al., 2010; Osickova et al., 2020). This is not a broadly applicable technique for KFA enzymes and as such will not be further elaborated on. Here we briefly introduce several proven strategies for measuring the activity of KFA hydrolases or transferases (Figure 6).

A commonly used approach to assaying KFA hydrolase activity is to incubate the enzyme of interest with a short peptide containing a KFA-modified lysine. After quenching the reaction, the products can be analyzed using HPLC or LC/MS (Figure 6B). For standard HPLC detection, the peptide ideally should have a strong chromophore which can be something as simple as a tryptophan residue. While this approach has been successfully implemented to study sirtuinss, HDACs, and NMTs, it could theoretically be applied for studying RtxC enzymes (Feldman et al., 2013; Teng et al., 2015; Aramsangtienchai et al., 2016; Cao et al., 2019; Kosciuk et al., 2020). One of the benefits of this assay is that a peptide closely resembling the physiological protein substrate can be used. It allows for determination of an enzyme’s substrate specificity by assaying peptides of various amino acid sequences. Additionally, tandem MS can be used to confirm the site of modification. Drawbacks for this approach include the need for HPLC or LC/MS instruments and relatively low throughput.

Acyl-peptide based assays for KFA hydrolases can also be analyzed with a fluorescence readout (Figure 6C). In this approach, peptides containing amino acids of choice with the acyl lysine at the C-terminal end followed by a fluorescent moiety such as 7-amino-4-methylcoumarin moiety (AMC) which will only fluoresce if released from the peptide (Young Hong et al., 2019). After incubation with enzyme the reaction is quenched by adding trypsin which cleaves amide bonds after lysines. Increased KFA hydrolase activity can thus be measured by increased fluorescence. This assay can be done quickly in a 96-well plate which allows researchers to test many different conditions at once and in replicates. This assay is well suited to screening libraries of compounds for enzyme inhibitors or activators. IC_50_ and EC_50_ values can be easily calculated by relating fluorescence values to the concentration of inhibitor or activator in the well. The drawback of this assay is the potential difficulty in synthesizing the appropriate peptide substrates. Furthermore, there are no amino acids on the C-terminal of the acyl-lysine, limiting the degree to which sequence specificity of a given enzyme can be ascertained. A similar technique is to modify a lysine with a fluorescent acyl group and to incorporate a fluorescence quenching moiety into the peptide. Hydrolysis separates the fluorophore from the quenching moiety and results in increased fluorescence. While this assay is also fast, the acyl group is necessarily bulky and may not have broad applicability (Kutil et al., 2019).

A promising novel technique for studying protein lipidation is acylation-coupled lipophilic induction of polarization (Acyl-cLIP) (Lanyon-Hogg et al., 2019). In this assay, an enzymatic protein lipidation reaction is carried out on a fluorescein tagged peptide in the presence of detergent micelles. Changes in lipidation affect the peptides affinity for the micelles. When bound to the micelles, the fluorescein has decreased molecular tumbling and a corresponding increase in polarized fluorescence emission. The change in polarized fluorescence can be detected with fluorescence anisotropy measurements and this change can be attributed to the lipidation status of the peptide (Figure 6D). Drawbacks of this assay are the same as other peptide-based assays mentioned above. Benefits of this assay include a high throughput for kinetics and inhibitor studies, the ability to assay both lipid transferase and hydrolase enzymes, and a universal applicability to any type of lipid modification.

### Measuring KFA With Fatty Acyl Probes

**FIGURE 7:**
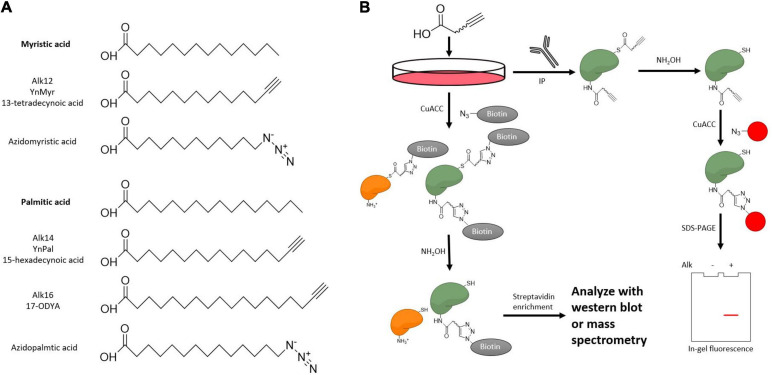
Metabolic probes to examine KFA on a substrate protein. **(A)** Structure of KFA probes. Bioorthogonal probes for KFA can mimic myristic acid or palmitic acid. Both azide and alkyne probes are applicable though alkyne probes more closely resemble the endogenous fatty acids. **(B)** Metabolic labeling of KFA proteins can be analyzed with CuACC. Alkyne labeled proteins can be modified with fluorophores or affinity tags like biotin to analyze KFA levels with in-gel fluorescence, western blot, or mass spectrometry.

If a target protein can be readily purified in large quantities, endogenous KFA levels can be analyzed with mass spectrometry, such as for APQ0 (Schey et al., 2010). However, this approach is not broadly applicable for reasons outlined above. More commonly used tools for studying levels of KFA on a target protein involve the addition of exogenous fatty acid analogs (Figure 7A). An important caveat for all approaches that involve the incorporation of fatty acids is that more than just lysines can be fatty acylated. Cysteine palmitoylation and glycine myristoylation are both abundant modifications that involve fatty acyl groups and must be ruled out as the source of fatty acylation signal. Cysteine palmitoylation can be hydrolyzed by hydroxylamine while lysine acylations cannot. Treating a fatty-acylated sample with hydroxylamine can thus eliminate an experimental signal from cysteine fatty-acylation. Glycine myristoylation cannot be removed with hydroxylamine but does requires a glycine at the most N-terminal position so a cursory examination of the protein’s sequence can reveal if the modification is possible.

One class of exogenous fatty acyl probes is radioactive-isotope-labeled fatty acids. Cells treated with radioactive fatty acids can then form radioactive fatty acyl-CoA for use by KFA transferases. A protein of interest can then be purified and monitored for radioactivity to assay for fatty acylation (Bursten et al., 1988). ^3^H- and ^14^C-labeled fatty acids are the best choice for this application as the isotopes can be incorporated into the fatty acid without modifying the structure. However, these isotopes have relatively low signal and must be monitored for a long time.

Clickable fatty acid analogs have proven to be a more powerful tool for studying KFA. After treating cells with these probes, proteins are modified with fluorophores or biotin via click chemistry to track levels of fatty acylation using fluorescence scanning, western blot, or various mass spectrometry approaches (Figure 7B). While azido fatty acids have been implemented for the study of cysteine palmitoylation, alkyne fatty acids have lower background and more closely resemble the structure of the endogenous fatty acids (Speers and Cravatt, 2004). There is a suite of fatty acyl alkyne probes that analogize natural fatty acids (Figure 7A). In addition to analyzing a protein of interest, clickable fatty acid probes can help identify substrates of KFA transferases or hydrolases as has been done for IcsB and HDAC11 (Liu et al., 2018; Cao et al., 2019). In this SILAC (stable isotope labeling with amino acids in cell culture) experiment, cells are cultured in media containing normal or isotopically heavy amino acids. KFA modulating enzymes are then overexpressed or suppressed and cells are treated with fatty acid alkyne probes. After probe incubation, cells are lysed and lysates from heavy and light media are mixed. Alkyne-labeled proteins are modified with biotin via click chemistry then treated with hydroxylamine to remove cysteine palmitoylation signal. The remaining biotinylated proteins are enriched with streptavidin and analyzed via MS. Further proteomic analysis in this way is necessary to fully appreciate the scope of KFA during various biological processes.

## Summary and Future Questions

Since the initial discovery of KFA on IL-1α, significant progress has been made in studying KFA. This is especially true of the last 10 or so years as several enzymes were identified to add or remove KFA. Additionally, implementation of clickable probes has allowed for the identification of many new proteins with KFA and has hastened the pace of discovery. We now know that KFA can regulate a variety of biological processes such as protein secretion, tumorigenesis, and immune signaling. The knowledge gained surrounding KFA can now serve as a basis for further exploration.

Multiple species of pathogenic bacteria utilize KFA to enhance their pathogenesis. While mammalian cells are not yet known to have KFA transferases that act on a broad range of substrates, the presence of multiple enzymes with strong KFA hydrolase activity suggests an evolutionarily beneficial role for this activity. Is it possible that during certain bacterial infections KFA hydrolase activity serves a protective role against toxins like RID and IcsB? Is it possible that KFA hydrolase enzymes could remove RTX toxin KFA to inactivate the pore-forming toxin?

Additional lines of future study are myriad. In addition to bacteria, could other pathogens modulate KFA during infection? In addition to infection, KFA has already been shown to modulate tumor phenotypes. What other diseases could be regulated by KFA? Can KFA guide the creation of therapeutics to counteract these diseases? Addressing these questions, will require identifying more proteins modified by KFA and additional enzymes that regulate this modification, as well as the development of small molecules targeting the regulatory enzymes. Future work needs to address these issues to fully understand the biological impact of KFA.

## Author Contributions

GK and HL surveyed the literature and wrote the manuscript. Both authors contributed to the article and approved the submitted version.

## Conflict of Interest

HL was a founder and consultant for Sedec Therapeutics. The remaining author declares that the research was conducted in the absence of any commercial or financial relationships that could be construed as a potential conflict of interest.

## References

[B1] Afonina I. S. Muller C. Martin S. J. Beyaert R. (2015). Proteolytic processing of Interleukin-1 family cytokines: variations on a common theme. *Immunity* 42 991–1004. 10.1016/j.immuni.2015.06.003 26084020

[B2] Allaoui A. Mounier J. Prevost M. C. Sansonetti P. J. Parsot C. (1992). icsB: a *Shigella* flexneri virulence gene necessary for the lysis of protrusions during intercellular spread. *Mol. Microbiol.* 6 1605–1616. 10.1111/j.1365-2958.1992.tb00885.x 1495389

[B3] Aramsangtienchai P. Spiegelman N. A. He B. Miller S. P. Dai L. Zhao Y. (2016). HDAC8 catalyzes the hydrolysis of long chain fatty acyl lysine. *ACS Chem. Biol.* 11 2685–2692. 10.1021/acschembio.6b00396 27459069 PMC5305809

[B4] Atapattu D. N. Czuprynski C. J. (2007). Mannheimia haemolytica leukotoxin binds to lipid rafts in bovine lymphoblastoid cells and is internalized in a dynamin-2- and clathrin-dependent manner. *Infect. Immun.* 75 4719–4727. 10.1128/IAI.00534-07 17682044 PMC2044511

[B5] Bagchi R. A. Ferguson B. S. Stratton M. S. Hu T. Cavasin M. A. Sun L. (2018). HDAC11 suppresses the thermogenic program of adipose tissue via BRD2. *JCI Insight* 3:e120159. 10.1172/jci.insight.120159 30089714 PMC6129125

[B6] Balashova N. V. Shah C. Patel J. K. Megalla S. Kachlany S. C. (2009). Aggregatibacter actinomycetemcomitans LtxC is required for leukotoxin activity and initial interaction between toxin and host cells. *Gene* 443 42–47. 10.1016/j.gene.2009.05.002 19450669

[B7] Ball L. E. Garland D. L. Crouch R. K. Schey K. L. (2004). Post-translational modifications of aquaporin 0 (AQP0) in the normal human lens: spatial and temporal occurrence. *Biochemistry* 43 9856–9865. 10.1021/bi0496034 15274640

[B8] Basar T. Havlicek V. Bezouskova S. Hackett M. Sebo P. (2001). Acylation of lysine 983 is sufficient for toxin activity of *Bordetella pertussis* adenylate cyclase. Substitutions of alanine 140 modulate acylation site selectivity of the toxin acyltransferase CyaC. *J. Biol. Chem.* 276 348–354. 10.1074/jbc.M006463200 11031260

[B9] Bellalou J. Sakamoto H. Ladant D. Geoffroy C. Ullmann A. (1990). Deletions affecting hemolytic and toxin activities of *Bordetella pertussis* adenylate cyclase. *Infect. Immun.* 58 3242–3247. 10.1128/IAI.58.10.3242-3247.1990 2401563 PMC313645

[B10] Benz R. (2016). Channel formation by RTX-toxins of pathogenic bacteria: basis of their biological activity. *Biochim. Biophys. Acta* 1858 526–537. 10.1016/j.bbamem.2015.10.025 26523409

[B11] Benz R. (2020). RTX-toxins. *Toxins (Basel)* 12 359. 10.3390/toxins12060359 32486155 PMC7354457

[B12] Berthiaume L. G. Beauchamp E. (2018). *Epigenetic Silencing of NMT2*, Patent US 20180208990A1. Edmonton CMA: Pacylex Pharmaceuticals.

[B13] Beuscher H. U. Colten H. R. (1988). Structure and function of membrane IL-1. *Mol. Immunol.* 25 1189–1199. 10.1016/0161-5890(88)90155-1 3265477

[B14] Biro M. Munoz M. A. Weninger W. (2014). Targeting Rho-GTPases in immune cell migration and inflammation. *Br. J. Pharmacol.* 171 5491–5506. 10.1111/bph.12658 24571448 PMC4282076

[B15] Bora-Singhal N. Mohankumar D. Saha B. Colin C. M. Lee J. Y. Martin M. W. (2020). Novel HDAC11 inhibitors suppress lung adenocarcinoma stem cell self-renewal and overcome drug resistance by suppressing Sox2. *Sci. Rep.* 10:4722. 10.1038/s41598-020-61295-6 32170113 PMC7069992

[B16] Bros M. Haas K. Moll L. Grabbe S. (2019). RhoA as a key regulator of innate and adaptive immunity. *Cells* 8:733. 10.3390/cells8070733 31319592 PMC6678964

[B17] Bursten S. L. Locksley R. M. Ryan J. L. Lovett D. H. (1988). Acylation of monocyte and glomerular mesangial cell proteins. Myristyl acylation of the interleukin 1 precursors. *J. Clin. Invest.* 82 1479–1488. 10.1172/JCI113755 3263392 PMC442712

[B18] Cao J. Sun L. Aramsangtienchai P. Spiegelman N. A. Zhang X. Huang W. (2019). HDAC11 regulates type I interferon signaling through defatty-acylation of SHMT2. *Proc. Natl. Acad. Sci. U.S.A.* 116 5487–5492. 10.1073/pnas.1815365116 30819897 PMC6431144

[B19] Chang A. R. Ferrer C. M. Mostoslavsky R. (2020). SIRT6, a mammalian deacylase with multitasking abilities. *Physiol. Rev.* 100 145–169. 10.1152/physrev.00030.2018 31437090 PMC7002868

[B20] Chen G. Huang P. Hu C. (2020). The role of SIRT2 in cancer: a novel therapeutic target. *Int. J. Cancer* 147 3297–3304. 10.1002/ijc.33118 32449165

[B21] Cordero C. L. Kudryashov D. S. Reisler E. Satchell K. J. (2006). The actin cross-linking domain of the *Vibrio cholerae* RTX toxin directly catalyzes the covalent cross-linking of actin. *J. Biol. Chem.* 281 32366–32374. 10.1074/jbc.M605275200 16954226 PMC2255562

[B22] Cuthbertson C. R. Arabzada Z. Bankhead A. III Kyani A. Neamati N. (2021). A review of small-molecule inhibitors of one-carbon enzymes: SHMT2 and MTHFD2 in the spotlight. *ACS Pharmacol. Transl. Sci.* 4 624–646. 10.1021/acsptsci.0c00223 33860190 PMC8033767

[B23] Deubzer H. E. Schier M. C. Oehme I. Lodrini M. Haendler B. Sommer A. (2013). HDAC11 is a novel drug target in carcinomas. *Int. J. Cancer* 132 2200–2208. 10.1002/ijc.27876 23024001

[B24] Dian C. Perez-Dorado I. Riviere F. Asensio T. Legrand P. Ritzefeld M. (2020). High-resolution snapshots of human N-myristoyltransferase in action illuminate a mechanism promoting N-terminal Lys and Gly myristoylation. *Nat. Commun.* 11:1132. 10.1038/s41467-020-14847-3 32111831 PMC7048800

[B25] Dominy J. E. Jr. Lee Y. Jedrychowski M. P. Chim H. Jurczak M. J. Camporez J. P. (2012). The deacetylase Sirt6 activates the acetyltransferase GCN5 and suppresses hepatic gluconeogenesis. *Mol. Cell* 48 900–913. 10.1016/j.molcel.2012.09.030 23142079 PMC3534905

[B26] El-Azami-El-Idrissi M. Bauche C. Loucka J. Osicka R. Sebo P. Ladant D. (2003). Interaction of *Bordetella pertussis* adenylate cyclase with CD11b/CD18: role of toxin acylation and identification of the main integrin interaction domain. *J. Biol. Chem.* 278 38514–38521. 10.1074/jbc.M304387200 12885782

[B27] Farazi T. A. Waksman G. Gordon J. I. (2001). The biology and enzymology of protein N-myristoylation. *J. Biol. Chem.* 276 39501–39504. 10.1074/jbc.R100042200 11527981

[B28] Feldman J. L. Baeza J. Denu J. M. (2013). Activation of the protein deacetylase SIRT6 by long-chain fatty acids and widespread deacylation by mammalian sirtuins. *J. Biol. Chem.* 288 31350–31356. 10.1074/jbc.C113.511261 24052263 PMC3829447

[B29] Ferrara G. Benzi A. Sturla L. Marubbi D. Frumento D. Spinelli S. (2020). Sirt6 inhibition delays the onset of experimental autoimmune encephalomyelitis by reducing dendritic cell migration. *J. Neuroinflammation* 17:228. 10.1186/s12974-020-01906-1 32736564 PMC7393881

[B30] Fiskus W. Coothankandaswamy V. Chen J. Ma H. Ha K. Saenz D. T. (2016). SIRT2 deacetylates and inhibits the peroxidase activity of Peroxiredoxin-1 to sensitize breast cancer cells to oxidant stress-inducing agents. *Cancer Res.* 76 5467–5478. 10.1158/0008-5472.CAN-16-0126 27503926 PMC5345574

[B31] Frey J. (2019). RTX toxins of animal pathogens and their role as antigens in vaccines and diagnostics. *Toxins (Basel)* 11:719. 10.3390/toxins11120719 31835534 PMC6950323

[B32] Gai W. Li H. Jiang H. Long Y. Liu D. (2016). Crystal structures of SIRT3 reveal that the alpha2-alpha3 loop and alpha3-helix affect the interaction with long-chain acyl lysine. *FEBS Lett.* 590 3019–3028. 10.1002/1873-3468.12345 27501476

[B33] Gao L. Cueto M. A. Asselbergs F. Atadja P. (2002). Cloning and functional characterization of HDAC11, a novel member of the human histone deacetylase family. *J. Biol. Chem.* 277 25748–25755. 10.1074/jbc.M111871200 11948178

[B34] Gillingham A. K. Munro S. (2007). The small G proteins of the Arf family and their regulators. *Annu. Rev. Cell Dev. Biol.* 23 579–611. 10.1146/annurev.cellbio.23.090506.123209 17506703

[B35] Glozak M. A. Seto E. (2009). Acetylation/deacetylation modulates the stability of DNA replication licensing factor Cdt1. *J. Biol. Chem.* 284 11446–11453. 10.1074/jbc.M809394200 19276081 PMC2670150

[B36] Goldberg J. (1998). Structural basis for activation of ARF GTPase: mechanisms of guanine nucleotide exchange and GTP-myristoyl switching. *Cell* 95 237–248. 10.1016/s0092-8674(00)81754-7 9790530

[B37] Gong D. Zeng Z. Yi F. Wu J. (2019). Inhibition of histone deacetylase 11 promotes human liver cancer cell apoptosis. *Am. J. Transl. Res.* 11 983–990. 30899397 PMC6413277

[B38] Greene N. P. Crow A. Hughes C. Koronakis V. (2015). Structure of a bacterial toxin-activating acyltransferase. *Proc. Natl. Acad. Sci. U.S.A.* 112 E3058–E3066. 10.1073/pnas.1503832112 26016525 PMC4466738

[B39] Gregoretti I. V. Lee Y. M. Goodson H. V. (2004). Molecular evolution of the histone deacetylase family: functional implications of phylogenetic analysis. *J. Mol. Biol.* 338 17–31. 10.1016/j.jmb.2004.02.006 15050820

[B40] Guin S. Ru Y. Wynes M. W. Mishra R. Lu X. Owens C. (2013). Contributions of KRAS and RAL in non-small-cell lung cancer growth and progression. *J. Thorac. Oncol.* 8 1492–1501. 10.1097/JTO.0000000000000007 24389431 PMC3934792

[B41] Guo F. (2021). RhoA and Cdc42 in T cells: are they targetable for T cell-mediated inflammatory diseases? *Precis. Clin. Med.* 4 56–61. 10.1093/pcmedi/pbaa039 33842837 PMC8023016

[B42] Hackett M. Guo L. Shabanowitz J. Hunt D. F. Hewlett E. L. (1994). Internal lysine palmitoylation in adenylate cyclase toxin from *Bordetella pertussis*. *Science* 266 433–435. 10.1126/science.7939682 7939682

[B43] Hackett M. Walker C. B. Guo L. Gray M. C. Van Cuyk S. Ullmann A. (1995). Hemolytic, but not cell-invasive activity, of adenylate cyclase toxin is selectively affected by differential fatty-acylation in *Escherichia coli*. *J. Biol. Chem.* 270 20250–20253. 10.1074/jbc.270.35.20250 7657593

[B44] He B. Hu J. Zhang X. Lin H. (2014). Thiomyristoyl peptides as cell-permeable Sirt6 inhibitors. *Org. Biomol. Chem.* 12 7498–7502. 10.1039/c4ob00860j 25163004 PMC4161628

[B45] Herlax V. Mate S. Rimoldi O. Bakas L. (2009). Relevance of fatty acid covalently bound to *Escherichia coli* alpha-hemolysin and membrane microdomains in the oligomerization process. *J. Biol. Chem.* 284 25199–25210. 10.1074/jbc.M109.009365 19596862 PMC2757223

[B46] Hong J. Y. Jing H. Price I. R. Cao J. Bai J. J. Lin H. (2020). Simultaneous Inhibition of SIRT2 deacetylase and defatty-acylase activities via a PROTAC strategy. *ACS Med. Chem. Lett.* 11 2305–2311. 10.1021/acsmedchemlett.0c00423 33214845 PMC7667848

[B47] Hong J. Y. Price I. R. Bai J. J. Lin H. (2019). A glycoconjugated SIRT2 inhibitor with aqueous solubility allows structure-based design of SIRT2 inhibitors. *ACS Chem. Biol.* 14 1802–1810. 10.1021/acschembio.9b00384 31373792 PMC6942458

[B48] Ismail V. S. Mosely J. A. Tapodi A. Quinlan R. A. Sanderson J. M. (2016). The lipidation profile of aquaporin-0 correlates with the acyl composition of phosphoethanolamine lipids in lens membranes. *Biochim. Biophys. Acta* 1858 2763–2768. 10.1016/j.bbamem.2016.06.026 27378310

[B49] Issartel J. P. Koronakis V. Hughes C. (1991). Activation of *Escherichia coli* prohaemolysin to the mature toxin by acyl carrier protein-dependent fatty acylation. *Nature* 351 759–761. 10.1038/351759a0 2062368

[B50] Iwaki M. Ullmann A. Sebo P. (1995). Identification by in vitro complementation of regions required for cell-invasive activity of *Bordetella pertussis* adenylate cyclase toxin. *Mol. Microbiol.* 17 1015–1024. 10.1111/j.1365-2958.1995.mmi_17061015.x 8594322

[B51] Jiang H. Khan S. Wang Y. Charron G. He B. Sebastian C. (2013). SIRT6 regulates TNF-alpha secretion through hydrolysis of long-chain fatty acyl lysine. *Nature* 496 110–113. 10.1038/nature12038 23552949 PMC3635073

[B52] Jiang H. Zhang X. Chen X. Aramsangtienchai P. Tong Z. Lin H. (2018). Protein lipidation: occurrence, mechanisms, biological functions, and enabling technologies. *Chem. Rev.* 118 919–988. 10.1021/acs.chemrev.6b00750 29292991 PMC5985209

[B53] Jiang H. Zhang X. Lin H. (2016). Lysine fatty acylation promotes lysosomal targeting of TNF-alpha. *Sci. Rep.* 6:24371. 10.1038/srep24371 27079798 PMC4832147

[B54] Jing H. Hu J. He B. Negron Abril Y. L. Stupinski J. Weiser K. (2016). A SIRT2-selective inhibitor promotes c-Myc oncoprotein degradation and exhibits broad anticancer activity. *Cancer Cell* 29 767–768. 10.1016/j.ccell.2016.04.005 27165747

[B55] Jing H. Zhang X. Wisner S. A. Chen X. Spiegelman N. A. Linder M. E. (2017). SIRT2 and lysine fatty acylation regulate the transforming activity of K-Ras4a. *Elife* 6:e32436. 10.7554/eLife.32436 29239724 PMC5745086

[B56] Kawahara T. L. Michishita E. Adler A. S. Damian M. Berber E. Lin M. (2009). SIRT6 links histone H3 lysine 9 deacetylation to NF-kappaB-dependent gene expression and organismal life span. *Cell* 136 62–74. 10.1016/j.cell.2008.10.052 19135889 PMC2757125

[B57] Kim B. Lee Y. Kim E. Kwak A. Ryoo S. Bae S. H. (2013). The Interleukin-1alpha precursor is biologically active and is likely a key alarmin in the IL-1 family of cytokines. *Front. Immunol.* 4:391. 10.3389/fimmu.2013.00391 24312098 PMC3834611

[B58] Knapp O. Maier E. Polleichtner G. Masin J. Sebo P. Benz R. (2003). Channel formation in model membranes by the adenylate cyclase toxin of *Bordetella pertussis*: effect of calcium. *Biochemistry* 42 8077–8084. 10.1021/bi034295f 12834359

[B59] Kosciuk T. Lin H. (2020). N-Myristoyltransferase as a glycine and lysine myristoyltransferase in cancer, immunity, and infections. *ACS Chem. Biol.* 15 1747–1758. 10.1021/acschembio.0c00314 32453941 PMC7841852

[B60] Kosciuk T. Price I. R. Zhang X. Zhu C. Johnson K. N. Zhang S. (2020). NMT1 and NMT2 are lysine myristoyltransferases regulating the ARF6 GTPase cycle. *Nat. Commun.* 11:1067. 10.1038/s41467-020-14893-x 32103017 PMC7044312

[B61] Kutil Z. Mikesova J. Zessin M. Meleshin M. Novakova Z. Alquicer G. (2019). Continuous activity assay for HDAC11 enabling reevaluation of HDAC inhibitors. *ACS Omega* 4 19895–19904. 10.1021/acsomega.9b02808 31788622 PMC6882135

[B62] Kutil Z. Novakova Z. Meleshin M. Mikesova J. Schutkowski M. Barinka C. (2018). Histone deacetylase 11 is a fatty-acid deacylase. *ACS Chem. Biol.* 13 685–693. 10.1021/acschembio.7b00942 29336543

[B63] Lanyon-Hogg T. Ritzefeld M. Sefer L. Bickel J. K. Rudolf A. F. Panyain N. (2019). Acylation-coupled lipophilic induction of polarisation (Acyl-cLIP): a universal assay for lipid transferase and hydrolase enzymes. *Chem. Sci.* 10 8995–9000. 10.1039/c9sc01785b 31762980 PMC6855259

[B64] Levental I. Lingwood D. Grzybek M. Coskun U. Simons K. (2010). Palmitoylation regulates raft affinity for the majority of integral raft proteins. *Proc. Natl. Acad. Sci. U.S.A.* 107 22050–22054. 10.1073/pnas.1016184107 21131568 PMC3009825

[B65] Lim K. B. Walker C. R. Guo L. Pellett S. Shabanowitz J. Hunt D. F. (2000). *Escherichia coli* alpha-hemolysin (HlyA) is heterogeneously acylated in vivo with 14-, 15-, and 17-carbon fatty acids. *J. Biol. Chem.* 275 36698–36702. 10.1074/jbc.C000544200 10978310

[B66] Linhartova I. Bumba L. Masin J. Basler M. Osicka R. Kamanova J. (2010). RTX proteins: a highly diverse family secreted by a common mechanism. *FEMS Microbiol. Rev.* 34 1076–1112. 10.1111/j.1574-6976.2010.00231.x 20528947 PMC3034196

[B67] Liu W. Zhou Y. Peng T. Zhou P. Ding X. Li Z. (2018). N(epsilon)-fatty acylation of multiple membrane-associated proteins by *Shigella* IcsB effector to modulate host function. *Nat. Microbiol.* 3 996–1009. 10.1038/s41564-018-0215-6 30061757 PMC6466622

[B68] Liu Y. Zhang Y. Zhu K. Chi S. Wang C. Xie A. (2019). Emerging role of Sirtuin 2 in Parkinson’s disease. *Front Aging Neurosci.* 11:372. 10.3389/fnagi.2019.00372 31998119 PMC6965030

[B69] Locksley R. M. Killeen N. Lenardo M. J. (2001). The TNF and TNF receptor superfamilies: integrating mammalian biology. *Cell* 104 487–501. 10.1016/s0092-8674(01)00237-9 11239407

[B70] Ludwig A. Garcia F. Bauer S. Jarchau T. Benz R. Hoppe J. (1996). Analysis of the in vivo activation of hemolysin (HlyA) from *Escherichia coli*. *J. Bacteriol.* 178 5422–5430. 10.1128/jb.178.18.5422-5430.1996 8808931 PMC178361

[B71] Martin M. W. Lee J. Y. Lancia D. R. Jr. Ng P. Y. Han B. Thomason J. R. (2018). Discovery of novel N-hydroxy-2-arylisoindoline-4-carboxamides as potent and selective inhibitors of HDAC11. *Bioorg. Med. Chem. Lett.* 28 2143–2147. 10.1016/j.bmcl.2018.05.021 29776742

[B72] Martin T. D. Samuel J. C. Routh E. D. Der C. J. Yeh J. J. (2011). Activation and involvement of Ral GTPases in colorectal cancer. *Cancer Res.* 71 206–215. 10.1158/0008-5472.CAN-10-1517 21199803 PMC3062918

[B73] Masin J. Basler M. Knapp O. El-Azami-El-Idrissi M. Maier E. Konopasek I. (2005). Acylation of lysine 860 allows tight binding and cytotoxicity of Bordetella adenylate cyclase on CD11b-expressing cells. *Biochemistry* 44 12759–12766. 10.1021/bi050459b 16171390

[B74] Mathias R. T. Rae J. L. Baldo G. J. (1997). Physiological properties of the normal lens. *Physiol. Rev.* 77 21–50. 10.1152/physrev.1997.77.1.21 9016299

[B75] Mellini P. Itoh Y. Tsumoto H. Li Y. Suzuki M. Tokuda N. (2017). Potent mechanism-based sirtuin-2-selective inhibition by an in situ-generated occupant of the substrate-binding site, “selectivity pocket” and NAD(+)-binding site. *Chem. Sci.* 8 6400–6408. 10.1039/c7sc02738a 28989670 PMC5628579

[B76] Michishita E. Mccord R. A. Berber E. Kioi M. Padilla-Nash H. Damian M. (2008). SIRT6 is a histone H3 lysine 9 deacetylase that modulates telomeric chromatin. *Nature* 452 492–496. 10.1038/nature06736 18337721 PMC2646112

[B77] Michishita E. Mccord R. A. Boxer L. D. Barber M. F. Hong T. Gozani O. (2009). Cell cycle-dependent deacetylation of telomeric histone H3 lysine K56 by human SIRT6. *Cell Cycle* 8 2664–2666. 10.4161/cc.8.16.9367 19625767 PMC4474138

[B78] Moniot S. Forgione M. Lucidi A. Hailu G. S. Nebbioso A. Carafa V. (2017). Development of 1,2,4-oxadiazoles as potent and selective inhibitors of the human deacetylase Sirtuin 2: structure-activity relationship, X-Ray crystal structure, and anticancer activity. *J. Med. Chem.* 60 2344–2360. 10.1021/acs.jmedchem.6b01609 28240897

[B79] Moreno-Yruela C. Galleano I. Madsen A. S. Olsen C. A. (2018). Histone deacetylase 11 is an epsilon-N-Myristoyllysine hydrolase. *Cell Chem. Biol.* 25 849–856.e8. 10.1016/j.chembiol.2018.04.007 29731425

[B80] Mousnier A. Bell A. S. Swieboda D. P. Morales-Sanfrutos J. Perez-Dorado I. Brannigan J. A. (2018). Fragment-derived inhibitors of human N-myristoyltransferase block capsid assembly and replication of the common cold virus. *Nat. Chem.* 10 599–606. 10.1038/s41557-018-0039-2 29760414 PMC6015761

[B81] Niki Y. Yamada H. Kikuchi T. Toyama Y. Matsumoto H. Fujikawa K. (2004). Membrane-associated IL-1 contributes to chronic synovitis and cartilage destruction in human IL-1 alpha transgenic mice. *J. Immunol.* 172 577–584. 10.4049/jimmunol.172.1.577 14688369

[B82] Ntwasa M. Aapies S. Schiffmann D. A. Gay N. J. (2001). Drosophila embryos lacking N-myristoyltransferase have multiple developmental defects. *Exp. Cell Res.* 262 134–144. 10.1006/excr.2000.5086 11139338

[B83] Nunez-Alvarez Y. Suelves M. (2021). HDAC11: a multifaceted histone deacetylase with proficient fatty deacylase activity and its roles in physiological processes. *FEBS J.* 10.1111/febs.15895 [Epub ahead of print]. 33891374

[B84] Ogawa M. Yoshimori T. Suzuki T. Sagara H. Mizushima N. Sasakawa C. (2005). Escape of intracellular *Shigella* from autophagy. *Science* 307 727–731. 10.1126/science.1106036 15576571

[B85] Osickova A. Balashova N. Masin J. Sulc M. Roderova J. Wald T. (2018). Cytotoxic activity of Kingella kingae RtxA toxin depends on post-translational acylation of lysine residues and cholesterol binding. *Emerg. Microbes Infect.* 7:178. 10.1038/s41426-018-0179-x 30405113 PMC6221878

[B86] Osickova A. Khaliq H. Masin J. Jurnecka D. Sukova A. Fiser R. (2020). Acyltransferase-mediated selection of the length of the fatty acyl chain and of the acylation site governs activation of bacterial RTX toxins. *J. Biol. Chem.* 295 9268–9280. 10.1074/jbc.RA120.014122 32461253 PMC7363117

[B87] Ostolaza H. Gonzalez-Bullon D. Uribe K. B. Martin C. Amuategi J. Fernandez-Martinez X. (2019). Membrane permeabilization by pore-forming RTX Toxins: what kind of lesions do these toxins form? *Toxins (Basel)* 11:354. 10.3390/toxins11060354 31216745 PMC6628442

[B88] Outeiro T. F. Kontopoulos E. Altmann S. M. Kufareva I. Strathearn K. E. Amore A. M. (2007). Sirtuin 2 inhibitors rescue alpha-synuclein-mediated toxicity in models of Parkinson’s disease. *Science* 317 516–519. 10.1126/science.1143780 17588900

[B89] Pan P. W. Feldman J. L. Devries M. K. Dong A. Edwards A. M. Denu J. M. (2011). Structure and biochemical functions of SIRT6. *J. Biol. Chem.* 286 14575–14587. 10.1074/jbc.M111.218990 21362626 PMC3077655

[B90] Parenti M. D. Grozio A. Bauer I. Galeno L. Damonte P. Millo E. (2014). Discovery of novel and selective SIRT6 inhibitors. *J. Med. Chem.* 57 4796–4804. 10.1021/jm500487d 24785705

[B91] Parsot C. (2009). *Shigella* type III secretion effectors: how, where, when, for what purposes? *Curr. Opin. Microbiol.* 12 110–116. 10.1016/j.mib.2008.12.002 19157960

[B92] Pei J. Grishin N. V. (2009). The Rho GTPase inactivation domain in *Vibrio cholerae* MARTX toxin has a circularly permuted papain-like thiol protease fold. *Proteins* 77 413–419. 10.1002/prot.22447 19434753 PMC3688474

[B93] Priestle J. P. Schar H. P. Grutter M. G. (1989). Crystallographic refinement of interleukin 1 beta at 2.0 A resolution. *Proc. Natl. Acad. Sci. U.S.A.* 86 9667–9671. 10.1073/pnas.86.24.9667 2602367 PMC298562

[B94] Rabl J. (2020). BRCA1-A and BRISC: multifunctional molecular machines for ubiquitin signaling. *Biomolecules* 10:1503. 10.3390/biom10111503 33142801 PMC7692841

[B95] Rabl J. Bunker R. D. Schenk A. D. Cavadini S. Gill M. E. Abdulrahman W. (2019). Structural basis of BRCC36 function in DNA repair and immune regulation. *Mol. Cell* 75 483–497.e9. 10.1016/j.molcel.2019.06.002 31253574 PMC6695476

[B96] Ren X. Gelinas A. D. Von Carlowitz I. Janjic N. Pyle A. M. (2017). Structural basis for IL-1alpha recognition by a modified DNA aptamer that specifically inhibits IL-1alpha signaling. *Nat. Commun.* 8:810. 10.1038/s41467-017-00864-2 28993621 PMC5634487

[B97] Resh M. D. (1999). Fatty acylation of proteins: new insights into membrane targeting of myristoylated and palmitoylated proteins. *Biochim. Biophys. Acta* 1451 1–16. 10.1016/s0167-4889(99)00075-0 10446384

[B98] Resh M. D. (2006). Trafficking and signaling by fatty-acylated and prenylated proteins. *Nat. Chem. Biol.* 2 584–590. 10.1038/nchembio834 17051234

[B99] Ringel A. E. Roman C. Wolberger C. (2014). Alternate deacylating specificities of the archaeal sirtuins Sir2Af1 and Sir2Af2. *Protein Sci.* 23 1686–1697. 10.1002/pro.2546 25200501 PMC4253809

[B100] Rumpf T. Schiedel M. Karaman B. Roessler C. North B. J. Lehotzky A. (2015). Selective Sirt2 inhibition by ligand-induced rearrangement of the active site. *Nat. Commun.* 6:6263. 10.1038/ncomms7263 25672491 PMC4339887

[B101] Salah Ud-Din A. I. Tikhomirova A. Roujeinikova A. (2016). Structure and functional diversity of GCN5-Related N-Acetyltransferases (GNAT). *Int. J. Mol. Sci.* 17:1018. 10.3390/ijms17071018 27367672 PMC4964394

[B102] Satchell K. J. (2007). MARTX, multifunctional autoprocessing repeats-in-toxin toxins. *Infect. Immun.* 75 5079–5084. 10.1128/IAI.00525-07 17646359 PMC2168290

[B103] Satchell K. J. (2011). Structure and function of MARTX toxins and other large repetitive RTX proteins. *Annu. Rev. Microbiol.* 65 71–90. 10.1146/annurev-micro-090110-102943 21639783

[B104] Schey K. L. Gutierrez D. B. Wang Z. Wei J. Grey A. C. (2010). Novel fatty acid acylation of lens integral membrane protein aquaporin-0. *Biochemistry* 49 9858–9865. 10.1021/bi101415w 20942504 PMC3831505

[B105] Schiedel M. Herp D. Hammelmann S. Swyter S. Lehotzky A. Robaa D. (2018). Chemically induced degradation of Sirtuin 2 (Sirt2) by a proteolysis targeting chimera (PROTAC) based on sirtuin rearranging ligands (SirReals). *J. Med. Chem.* 61 482–491. 10.1021/acs.jmedchem.6b01872 28379698

[B106] Schlott A. C. Holder A. A. Tate E. W. (2018). N-Myristoylation as a drug target in malaria: exploring the role of N-Myristoyltransferase substrates in the inhibitor mode of action. *ACS Infect. Dis.* 4 449–457. 10.1021/acsinfecdis.7b00203 29363940

[B107] Selvakumar P. Lakshmikuttyamma A. Shrivastav A. Das S. B. Dimmock J. R. Sharma R. K. (2007). Potential role of N-myristoyltransferase in cancer. *Prog. Lipid Res.* 46 1–36. 10.1016/j.plipres.2006.05.002 16846646

[B108] Sheahan K. L. Satchell K. J. (2007). Inactivation of small Rho GTPases by the multifunctional RTX toxin from *Vibrio cholerae*. *Cell Microbiol.* 9 1324–1335. 10.1111/j.1462-5822.2006.00876.x 17474905 PMC2258554

[B109] Simanshu D. K. Nissley D. V. Mccormick F. (2017). RAS proteins and their regulators in human disease. *Cell* 170 17–33. 10.1016/j.cell.2017.06.009 28666118 PMC5555610

[B110] Sindhu Kumari S. Gupta N. Shiels A. Fitzgerald P. G. Menon A. G. Mathias R. T. (2015). Role of Aquaporin 0 in lens biomechanics. *Biochem. Biophys. Res. Commun.* 462 339–345. 10.1016/j.bbrc.2015.04.138 25960294 PMC4461499

[B111] Sociali G. Magnone M. Ravera S. Damonte P. Vigliarolo T. Von Holtey M. (2017). Pharmacological Sirt6 inhibition improves glucose tolerance in a type 2 diabetes mouse model. *FASEB J.* 31 3138–3149. 10.1096/fj.201601294R 28386046 PMC6137498

[B112] Son S. I. Cao J. Zhu C. L. Miller S. P. Lin H. (2019). Activity-guided design of HDAC11-specific inhibitors. *ACS Chem. Biol.* 14 1393–1397. 10.1021/acschembio.9b00292 31264832 PMC6893910

[B113] Son S. I. Su D. Ho T. T. Lin H. (2020). Garcinol is an HDAC11 inhibitor. *ACS Chem. Biol.* 15 2866–2871. 10.1021/acschembio.0c00719 33034447 PMC7857146

[B114] Speers A. E. Cravatt B. F. (2004). Profiling enzyme activities in vivo using click chemistry methods. *Chem. Biol.* 11 535–546. 10.1016/j.chembiol.2004.03.012 15123248

[B115] Spiegelman N. A. Price I. R. Jing H. Wang M. Yang M. Cao J. (2018). Direct comparison of SIRT2 inhibitors: potency, specificity, activity-dependent inhibition, and on-target anticancer activities. *ChemMedChem* 13 1890–1894. 10.1002/cmdc.201800391 30058233 PMC6402572

[B116] Spiegelman N. A. Zhang X. Jing H. Cao J. Kotliar I. B. Aramsangtienchai P. (2019). SIRT2 and lysine fatty acylation regulate the activity of RalB and cell migration. *ACS Chem. Biol.* 14 2014–2023. 10.1021/acschembio.9b00492 31433161 PMC6893912

[B117] Stanley P. Packman L. C. Koronakis V. Hughes C. (1994). Fatty acylation of two internal lysine residues required for the toxic activity of *Escherichia coli* hemolysin. *Science* 266 1992–1996. 10.1126/science.7801126 7801126

[B118] Stevenson F. T. Bursten S. L. Fanton C. Locksley R. M. Lovett D. H. (1993). The 31-kDa precursor of interleukin 1 alpha is myristoylated on specific lysines within the 16-kDa N-terminal propiece. *Proc. Natl. Acad. Sci. U.S.A.* 90 7245–7249. 10.1073/pnas.90.15.7245 8346241 PMC47113

[B119] Stevenson F. T. Bursten S. L. Locksley R. M. Lovett D. H. (1992). Myristyl acylation of the tumor necrosis factor alpha precursor on specific lysine residues. *J. Exp. Med.* 176 1053–1062. 10.1084/jem.176.4.1053 1402651 PMC2119375

[B120] Sun L. Marin De Evsikova C. Bian K. Achille A. Telles E. Pei H. (2018a). Programming and regulation of metabolic homeostasis by HDAC11. *EBioMedicine* 33 157–168. 10.1016/j.ebiom.2018.06.025 29958910 PMC6085537

[B121] Sun L. Telles E. Karl M. Cheng F. Luetteke N. Sotomayor E. M. (2018b). Loss of HDAC11 ameliorates clinical symptoms in a multiple sclerosis mouse model. *Life Sci. Alliance* 1:e201800039. 10.26508/lsa.201800039 30456376 PMC6238389

[B122] Tecleab A. Zhang X. Sebti S. M. (2014). Ral GTPase down-regulation stabilizes and reactivates p53 to inhibit malignant transformation. *J. Biol. Chem.* 289 31296–31309. 10.1074/jbc.M114.565796 25210032 PMC4223330

[B123] Teng Y. B. Jing H. Aramsangtienchai P. He B. Khan S. Hu J. (2015). Efficient demyristoylase activity of SIRT2 revealed by kinetic and structural studies. *Sci. Rep.* 5:8529. 10.1038/srep08529 25704306 PMC4894398

[B124] Tenhunen J. Kucera T. Huovinen M. Kublbeck J. Bisenieks E. Vigante B. (2021). Screening of SIRT6 inhibitors and activators: a novel activator has an impact on breast cancer cells. *Biomed. Pharmacother.* 138:111452. 10.1016/j.biopha.2021.111452 33684691 PMC12036750

[B125] Thole T. M. Lodrini M. Fabian J. Wuenschel J. Pfeil S. Hielscher T. (2017). Neuroblastoma cells depend on HDAC11 for mitotic cell cycle progression and survival. *Cell Death Dis.* 8:e2635. 10.1038/cddis.2017.49 28252645 PMC5386552

[B126] Toiber D. Erdel F. Bouazoune K. Silberman D. M. Zhong L. Mulligan P. (2013). SIRT6 recruits SNF2H to DNA break sites, preventing genomic instability through chromatin remodeling. *Mol. Cell* 51 454–468. 10.1016/j.molcel.2013.06.018 23911928 PMC3761390

[B127] Tong Z. Wang M. Wang Y. Kim D. D. Grenier J. K. Cao J. (2017). SIRT7 is an RNA-activated protein lysine deacylase. *ACS Chem. Biol.* 12 300–310. 10.1021/acschembio.6b00954 27997115 PMC5326686

[B128] Towler D. A. Adams S. P. Eubanks S. R. Towery D. S. Jackson-Machelski E. Glaser L. (1987). Purification and characterization of yeast myristoyl CoA:protein N-myristoyltransferase. *Proc. Natl. Acad. Sci. U.S.A.* 84 2708–2712. 10.1073/pnas.84.9.2708 3106975 PMC304727

[B129] Trent M. S. Worsham L. M. Ernst-Fonberg M. L. (1999). HlyC, the internal protein acyltransferase that activates hemolysin toxin: role of conserved histidine, serine, and cysteine residues in enzymatic activity as probed by chemical modification and site-directed mutagenesis. *Biochemistry* 38 3433–3439. 10.1021/bi982491u 10079090

[B130] Walden M. Tian L. Ross R. L. Sykora U. M. Byrne D. P. Hesketh E. L. (2019). Metabolic control of BRISC-SHMT2 assembly regulates immune signalling. *Nature* 570 194–199. 10.1038/s41586-019-1232-1 31142841 PMC6914362

[B131] Wang B. Dai T. Sun W. Wei Y. Ren J. Zhang L. (2021). Protein N-myristoylation: functions and mechanisms in control of innate immunity. *Cell. Mol. Immunol.* 18 878–888. 10.1038/s41423-021-00663-2 33731917 PMC7966921

[B132] Wang W. Fu L. Li S. Xu Z. Li X. (2017). Histone deacetylase 11 suppresses p53 expression in pituitary tumor cells. *Cell Biol. Int.* 41 1290–1295. 10.1002/cbin.10834 28782861

[B133] Wang Y. Yang J. Hong T. Chen X. Cui L. (2019). SIRT2: controversy and multiple roles in disease and physiology. *Ageing Res. Rev.* 55:100961. 10.1016/j.arr.2019.100961 31505260

[B134] Wassef J. S. Keren D. F. Mailloux J. L. (1989). Role of M cells in initial antigen uptake and in ulcer formation in the rabbit intestinal loop model of shigellosis. *Infect. Immun.* 57 858–863. 10.1128/IAI.57.3.858-863.1989 2645214 PMC313189

[B135] Welch R. A. (2001). RTX toxin structure and function: a story of numerous anomalies and few analogies in toxin biology. *Curr. Top. Microbiol. Immunol.* 257 85–111. 10.1007/978-3-642-56508-3_5 11417123

[B136] Woida P. J. Satchell K. J. F. (2020). The *Vibrio cholerae* MARTX toxin silences the inflammatory response to cytoskeletal damage before inducing actin cytoskeleton collapse. *Sci. Signal.* 13:eaaw9447. 10.1126/scisignal.aaw9447 31937566 PMC7309453

[B137] Worsham L. M. Trent M. S. Earls L. Jolly C. Ernst-Fonberg M. L. (2001). Insights into the catalytic mechanism of HlyC, the internal protein acyltransferase that activates *Escherichia coli* hemolysin toxin. *Biochemistry* 40 13607–13616. 10.1021/bi011032h 11695909

[B138] Yang H. Chen L. Sun Q. Yao F. Muhammad S. Sun C. (2021). The role of HDAC11 in obesity-related metabolic disorders: a critical review. *J. Cell. Physiol.* 236 5582–5591. 10.1002/jcp.30286 33481312

[B139] Yang S. H. Shrivastav A. Kosinski C. Sharma R. K. Chen M. H. Berthiaume L. G. (2005). N-myristoyltransferase 1 is essential in early mouse development. *J. Biol. Chem.* 280 18990–18995. 10.1074/jbc.M412917200 15753093

[B140] Yanginlar C. Logie C. (2018). HDAC11 is a regulator of diverse immune functions. *Biochim. Biophys. Acta* 1861 54–59. 10.1016/j.bbagrm.2017.12.002 29222071

[B141] You W. Rotili D. Li T. M. Kambach C. Meleshin M. Schutkowski M. (2017). Structural basis of Sirtuin 6 activation by synthetic small molecules. *Angew. Chem. Int. Ed. Engl.* 56 1007–1011. 10.1002/anie.201610082 27990725

[B142] You W. Zheng W. Weiss S. Chua K. F. Steegborn C. (2019). Structural basis for the activation and inhibition of Sirtuin 6 by quercetin and its derivatives. *Sci. Rep.* 9:19176. 10.1038/s41598-019-55654-1 31844103 PMC6914789

[B143] Young Hong J. Cao J. Lin H. (2019). Fluorogenic assays for the defatty-acylase activity of Sirtuins. *Methods Mol. Biol.* 2009 129–136. 10.1007/978-1-4939-9532-5_10 31152400 PMC6893880

[B144] Yuan M. Song Z. H. Ying M. D. Zhu H. He Q. J. Yang B. (2020). N-myristoylation: from cell biology to translational medicine. *Acta Pharmacol. Sin.* 41 1005–1015. 10.1038/s41401-020-0388-4 32203082 PMC7468318

[B145] Yuan Y. Zhao K. Yao Y. Liu C. Chen Y. Li J. (2019). HDAC11 restricts HBV replication through epigenetic repression of cccDNA transcription. *Antiviral Res.* 172:104619. 10.1016/j.antiviral.2019.104619 31600533

[B146] Zhang X. Khan S. Jiang H. Antonyak M. A. Chen X. Spiegelman N. A. (2016). Identifying the functional contribution of the defatty-acylase activity of SIRT6. *Nat. Chem. Biol.* 12 614–620. 10.1038/nchembio.2106 27322069 PMC4955683

[B147] Zhang X. Spiegelman N. A. Nelson O. D. Jing H. Lin H. (2017). SIRT6 regulates Ras-related protein R-Ras2 by lysine defatty-acylation. *Elife* 6:e25158. 10.7554/eLife.25158 28406396 PMC5391209

[B148] Zhao D. Mo Y. Li M. T. Zou S. W. Cheng Z. L. Sun Y. P. (2014). NOTCH-induced aldehyde dehydrogenase 1A1 deacetylation promotes breast cancer stem cells. *J. Clin. Invest.* 124 5453–5465. 10.1172/JCI76611 25384215 PMC4348941

[B149] Zheng H. Gupta V. Patterson-Fortin J. Bhattacharya S. Katlinski K. Wu J. (2013). A BRISC-SHMT complex deubiquitinates IFNAR1 and regulates interferon responses. *Cell Rep.* 5 180–193. 10.1016/j.celrep.2013.08.025 24075985 PMC3813903

[B150] Zhong L. D’urso A. Toiber D. Sebastian C. Henry R. E. Vadysirisack D. D. (2010). The histone deacetylase Sirt6 regulates glucose homeostasis via Hif1alpha. *Cell* 140 280–293. 10.1016/j.cell.2009.12.041 20141841 PMC2821045

[B151] Zhou W. Ni T. K. Wronski A. Glass B. Skibinski A. Beck A. (2016). The SIRT2 deacetylase stabilizes slug to control malignancy of basal-like breast cancer. *Cell Rep.* 17 1302–1317. 10.1016/j.celrep.2016.10.006 27783945 PMC5108094

[B152] Zhou Y. Huang C. Yin L. Wan M. Wang X. Li L. (2017). N(epsilon)-Fatty acylation of Rho GTPases by a MARTX toxin effector. *Science* 358 528–531. 10.1126/science.aam8659 29074776

[B153] Zhu A. Y. Zhou Y. Khan S. Deitsch K. W. Hao Q. Lin H. (2012). Plasmodium falciparum Sir2A preferentially hydrolyzes medium and long chain fatty acyl lysine. *ACS Chem. Biol.* 7 155–159. 10.1021/cb200230x 21992006 PMC3262940

[B154] Zychlinsky A. Prevost M. C. Sansonetti P. J. (1992). *Shigella* flexneri induces apoptosis in infected macrophages. *Nature* 358 167–169. 10.1038/358167a0 1614548

